# ELAV mediates circular RNA biogenesis in neurons

**DOI:** 10.1101/gad.352670.125

**Published:** 2025-09-01

**Authors:** Carlos Alfonso-Gonzalez, Mengjin Shi, Sakshi Gorey, Sarah Holec, Judit Carrasco, Michael Rauer, Stylianos Tsagkris, Fernando Mateos, Valérie Hilgers

**Affiliations:** 1 Max Planck Institute of Immunobiology and Epigenetics, 79108 Freiburg, Germany;; 2 Faculty of Biology, University of Freiburg, 79104 Freiburg, Germany

**Keywords:** circular RNAs, nervous system, RNA-binding proteins, ELAV, *Drosophila*, splicing, back-splicing, reverse complementary matches (RCMs), RNA processing, RNA binding motif

## Abstract

Circular RNAs (circRNAs) arise from back-splicing of precursor RNAs and accumulate in the nervous systems of animals, where they are thought to regulate gene expression and synaptic function. Here, we show that neuronal circRNA biosynthesis is mediated by the pan-neuronal RNA-binding protein ELAV. In *Drosophila* embryos, we characterized the circRNA landscape in normal and *elav* mutant neurons. We found that neuronal circRNAs are globally (>75%) depleted upon ELAV knockout, and induction of ELAV expression drives ectopic RNA circularization. In brain tissue, ELAV binds to pre-mRNA introns flanking putative circRNAs and decreases efficiency of linear splicing in favor of intron pairing at reverse complementary matches, inducing circularization. Together, our data demonstrate that ELAV directly modulates splicing decisions to generate the neuronal circRNA landscape.

## Abstract (precis)

In this study, Alfonso-Gonzalez et al. describe a role for RNA binding protein ELAV in the biogenesis of neuronal circRNAs in *Drosophila* embryos. ELAV binds to reverse complementary match sequences in the circRNA-flanking introns on the host precursor mRNA and promotes back-splicing and circularization over linear splicing, revealing an important role for ELAV in regulating neuronal splicing decisions.

Circular RNAs (circRNAs) are single-stranded RNA molecules covalently bound into a continuous loop, found ubiquitously across all domains of life (Liu and Chen 2022). In animals, circRNAs are expressed in tissue-specific and developmental stage-specific patterns, with a particularly high abundance in the nervous system (Salzman et al. 2012; Memczak et al. 2013; Wang et al. 2014; Patop et al. 2019). CircRNAs typically arise from genes linked to synaptic functions. Although their physiological functions remain much less well studied compared with the linear version of the gene, circRNAs have been shown to play important roles in various aspects of brain development and functionality, including neural stemness and neurodegeneration during aging, cognition, and plasticity (You et al. 2015; Gruner et al. 2016; Piwecka et al. 2017; Suenkel et al. 2020; Zimmerman et al. 2020; Morandell et al. 2024; Kirio et al. 2025). For instance, the loss of *Cdr1as* circRNA disrupts sensorimotor gating, a phenotype associated with several human neuropsychiatric disorders (Westholm et al. 2014; Rybak-Wolf et al. 2015). Moreover, circRNA expression is altered in multiple neuropathological conditions such as addiction and neurodegeneration, making them potential markers for these diseases (Dube et al. 2019; Dong et al. 2023). Mechanisms of action differ for distinct circRNAs (Liu and Chen 2022); some may act as regulators of individual genes (Pamudurti et al. 2022) or function globally as sponges for microRNAs and RNA-binding proteins (RBPs) (Hansen et al. 2013; Ashwal-Fluss et al. 2014), whereas others are translated into a functional protein (van Heesch et al. 2019; Weigelt et al. 2020).

CircRNA formation is a process in which a 5′ splice site is ligated “back” to a 3′ splice site located upstream, thereby forming a circular structure with a characteristic 3′–5′ phosphodiester bond at the back-splicing junction (BSJ) site (Chen and Yang 2015; Kristensen et al. 2019). Intron pairing is facilitated by secondary structures of flanking introns and promotes circRNA formation by bringing splice sites of distal exons into proximity with each other (Zhang et al. 2014; Ivanov et al. 2015). Both back-splicing and linear splicing use fundamental RNA processing components such as the spliceosome machinery and canonical splice sites (Ashwal-Fluss et al. 2014; Zhang et al. 2014, 2016; Starke et al. 2015; Li et al. 2019), which suggests that the two processes occur in a competitive manner. A subset of double-stranded RNA-binding domain-containing proteins, including immune factors NF90/NF110 (Li et al. 2017), the RNA helicase DHX9 (Aktaş et al. 2017), and the RNA-editing enzyme ADAR (Ivanov et al. 2015), bind complementary sequences flanking circRNAs and promote or inhibit back-splicing. Specific RBPs have been shown to influence circRNA expression in developmental transitions and in various cellular contexts. During epithelial–mesenchymal transition, the splicing factor Quaking regulates the formation of over one-third of abundant circRNAs through intron binding (Conn et al. 2015); in motor neurons, the RBP FUS interacts with the pre-mRNA to control the biogenesis of specific circRNAs (Errichelli et al. 2017), and the RNA processing factor NOVA2 promotes the biogenesis of numerous circRNAs in mouse cortical neurons (Knupp et al. 2021). The mechanisms governing circRNA regulation in vivo are yet to be fully elucidated; in particular, the intriguing prevalence of circRNAs in the nervous system indicates the involvement of a general mechanism or effector that shifts the balance between linear and circularizing splicing.

ELAV/Hu proteins are a family of highly conserved RBPs, of which at least one member is expressed in a nervous system-specific manner and used as marker of neuronal identity across animals (Yao et al. 1993; Pascale et al. 2008; Hilgers 2023). *elav*-null mutations are embryonic lethal, and ELAV activity is essential in neurogenesis for establishing and maintaining the neuronal RNA transcriptome via alternative splicing (AS) and alternative polyadenylation (APA) (Carrasco et al. 2020, 2022; Wei et al. 2020; Lee et al. 2021). This regulation occurs cotranscriptionally, with ELAV proteins targeting sequence elements at the 3′ ends or splice sites. ELAV's global role in RNA processing prompted the hypothesis that the RBP may be involved in cotranscriptional RNA circularization in the nervous system. Here, we unveil a previously unrecognized role of ELAV proteins as key mediators of circRNA expression in the nervous system and provide mechanistic insights into circRNA biogenesis.

## Results

### Characterization of the circRNA landscape in *Drosophila* embryos

**Figure 1.**
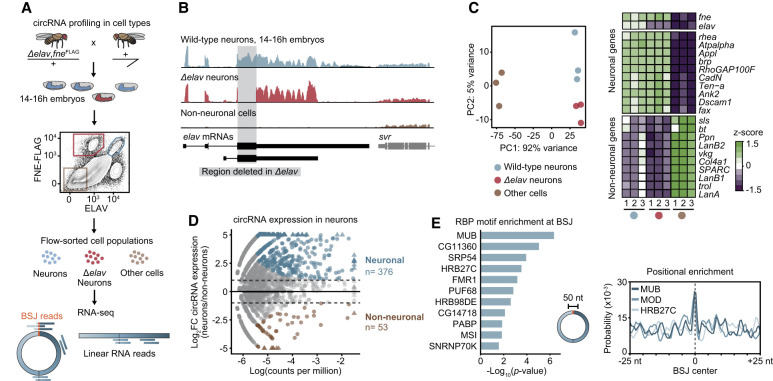
The circRNA landscape in *Drosophila* embryos. See also Supplemental Figures S1–S3 and Supplemental Table S1. (*A*) Experimental overview: Cells from embryonic progeny of Δ*elav,fne*^*FLAG*^ heterozygous flies (carrying an *elav*-null mutation recombined with a FLAG-tagged allele of the neuronal marker FNE) were FACS-sorted into three distinct populations: wild-type neurons (ELAV^+^, FLAG^+^), Δ*elav* mutant neurons (ELAV^−^, FLAG^+^), and nonneuronal cells (ELAV^−^, FLAG^−^). CircRNA expression was quantified from total RNA-seq data measuring reads spanning the back-splice junction (BSJ reads) unique to circRNAs. (*B*) Total RNA-seq tracks at the *elav* locus in the sorted cell populations. The loss of signal in the *elav* coding region in Δ*elav* neurons is highlighted. (*C*) Principal component analysis plot of gene expression across the embryonic cell populations. The heat map at the *right* indicates the top differentially expressed genes between neuronal and nonneuronal populations. Replicates and identities of cell populations are indicated with colored dots. (*D*) Differential circRNA expression in neurons compared with nonneuronal populations represented as a function of BSJ counts per million. Significantly enriched (neuronal) circRNAs [*P* < 0.05 and log(CPM) > −5.4, log_2_FC ≥ 1; blue] or depleted (nonneuronal) circRNAs (log_2_FC ≤ −1; brown) are highlighted. (*E*, *left*) RBP binding motifs significantly enriched at the BSJ (±25 nt) of neuronal circRNAs compared with the entire linearized circRNA sequence. (*Right*) Positional enrichment of binding motifs for three neuronal RBPs is indicated from the center of the back-splice junction.

To identify neuron-enriched circRNAs in vivo, we compared transcriptomes in distinct cell populations. We crossed heterozygous Δ*elav* mutant parental flies and collected embryonic progeny at 14–16 h after fertilization. The ELAV paralog FNE can partially compensate for ELAV functions in later embryonic stages (Carrasco et al. 2020, 2022); to account for this, in an independent experiment, we used Δ*elav*Δ*fne* mutants and a late stage, 18–20 h time point (Fig. 1A; Supplemental Fig. S1A). For each cross, embryonic tissues were dissociated into individual cells, fixed, fluorescently labeled, and flow-sorted into three distinct populations: wild-type neurons, mutant (Δ*elav* or Δ*elav*Δ*fne*) neurons, and pooled cells from all other embryonic tissues (“nonneurons”). RNA sequencing and gene expression profiling confirmed the purity (Fig. 1B; Supplemental Fig. S1B) of each population. Δ*elav* mutant neurons presented normal levels of neuronal marker genes and clustered with wild-type neurons in a principal component analysis, indicating that neuronal identity is largely unaffected in these cells (Fig. 1C).

To annotate and quantify circRNAs, we counted RNA-seq reads that span back-splice junctions (BSJs) identified from CIRI2 (Gao et al. 2018), including only circRNAs containing canonical splice signals. We treated bulk tissue samples from adult fly heads and 14–20 h embryos with RNaseR, a treatment that enriches for circRNAs, and compiled a circRNA reference transcriptome by pooling BSJ reads from all sample replicates (Supplemental Fig. S1C). We subsampled BSJ reads from each data set and assessed the number of identified circRNAs in each fraction for each cell population. Near saturation was achieved for circRNAs detected with five or more BSJ reads (Supplemental Fig. S1D); therefore, we set this value as a threshold for stringent circRNA identification and subsequent analyses, in line with good practice large benchmarking studies (Vromman et al. 2023).

Fifty-eight percent of the circRNAs identified (five or more BSJs) in the sorted cell populations were also present in the RNaseR-treated samples (Supplemental Fig. S1E,F), validating the accuracy of our annotation and indicating that isolating cell populations helps identify circRNAs that are expressed either lowly or in a tissue-specific manner. Comparing BSJ expression between neuronal and nonneuronal populations with stringent cutoffs, we identified ∼400 circRNAs specifically enriched in neurons at either developmental time (referred to here as “neuronal circRNAs”) (Fig. 1D; Supplemental Fig. S1G–J; Supplemental Table S1), with only 55 circRNAs depleted compared with other embryonic cells (“nonneuronal”). We used an additional circRNA annotation tool, CircSplice (Feng et al. 2019), to detect and quantify circRNAs in the individual cell populations. After filtering, CircSplice detected significantly fewer circRNAs than CIRI2 (Supplemental Fig. S2A–C); circRNA overlap and expression levels in the different cell populations, however, were highly consistent between the two methods (Supplemental Fig. S2D–H).

The majority of circRNAs enriched in the neuronal population are transcribed from genes associated with neuronal functions, with a notable enrichment for synaptic signaling and complex behavior (Supplemental Fig. S2I). This aligns with research in mouse tissues demonstrating that host genes of brain-specific circRNAs are highly enriched for synaptic proteins (You et al. 2015) and could indicate that circRNAs have a conserved function in the regulation of synaptic processes. BSJs are unique to circRNAs and may constitute binding sites for RBPs that regulate the circRNA in a manner distinct from that of the linear transcript. When we compared back-splice junction regions (BSJ ± 25 nt) of neuronal circRNAs with those of broadly expressed circRNAs, we observed a significant enrichment in motifs for various RBPs (Supplemental Fig. S2J,K). Interestingly, in neuronal circRNAs, when comparing the BSJ region with the linearized exon sequence, we found specific neuronal RBPs to be enriched, including mushroom body expressed (Mub), Musashi (Msi), and fragile X mental retardation protein (Fmr1). Some RBPs displayed exquisite positional enrichment at the center of the BSJ; i.e., the precise region discriminating circRNAs from their linear cognate (Fig. 1E). To test a possible involvement of some of these proteins in the expression of neuronal circRNAs, we analyzed RNA-seq data generated from fly brains in which RBPs were individually knocked down using RNAi (Sapiro et al. 2020). We found numerous circRNAs upregulated or downregulated upon knockdown of Adar, Fmr1, Hrb98DE, and Mub, which overlapped with neuronal circRNAs by up to 30% (circRNAs downregulated in *hrb98de* RNAi) (Supplemental Fig. S2L,M). This could indicate that several neuronal RBPs regulate the expression of neuronal circRNAs in a coordinated manner.

Individual circRNAs have been shown to act independently in cell type-specific gene expression regulation in different tissues (Khan et al. 2016; Legnini et al. 2017; Cherubini et al. 2019; Pamudurti et al. 2022; Zheng et al. 2022) and were proposed to act as tissue-specific RBP and microRNA “sponges” (Hansen et al. 2013; Westholm et al. 2014; Piwecka et al. 2017). Our results align with research showing that circRNAs represent molecular signatures of developing neurons (Ashwal-Fluss et al. 2014; Suenkel et al. 2020; Rybiczka-Tešulov et al. 2024) and reinforce the notion that circRNAs perform cellular functions distinct from those of linear transcripts.

### ELAV mediates neuronal circRNA expression

**Figure 2.**
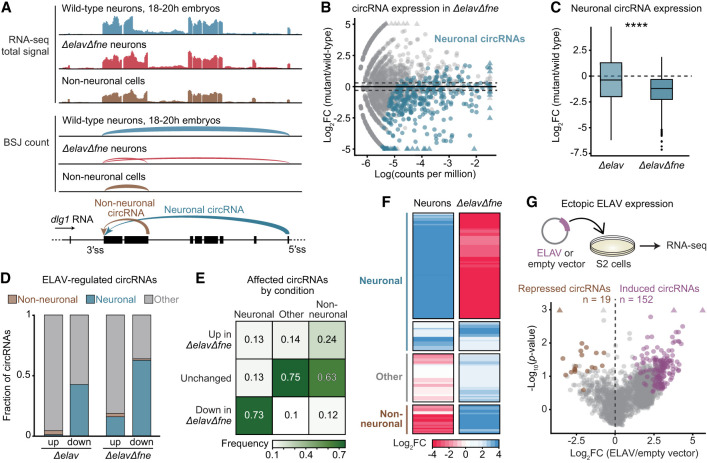
ELAV regulates neuronal circRNA expression. See also Supplemental Figures S2 and S3 and Supplemental Table S1. (*A*) Total RNA-seq signal tracks and representation of the BSJ count of a portion of the gene *discs large 1* (*dlg1*) in the sorted cell populations. Arrows in the gene model indicate the back-splicing events that produce the neuronal (blue) or nonneuronal (brown) circRNAs, respectively. (*B*) Differential circRNA expression in wild-type neurons compared with Δ*elav*Δ*fne* neurons, represented as a function of BSJ counts per million. Highlighted dots represent circRNAs classified as neuronal (376 neuronal circRNAs) (Fig. 1D). The dotted line indicates the abs(log_2_FC) ≥ 0.3 cutoff. (*C*) Differential circRNA expression in Δ*elav* and Δ*elav*Δ*fne* mutant neurons compared with wild-type neurons. (****) *P* < 0.0001 (two-tailed Welch's *t*-test). (*D*) Proportion of circRNAs with neuron-specific expression (neuronal, nonneuronal, and other) affected in Δ*elav* and Δ*elav*Δ*fne* neurons. CircRNAs were considered significantly affected (up or down) in mutant compared with wild-type neurons if abs(log_2_FC) ≥ 0.3 (Δ*elav*Δ*fne*) or abs(log_2_FC) ≥ 0.5 (Δ*elav*) and log(CPM) > −5.4. (*E*) Confusion matrix displaying the fraction of circRNAs with neuron-specific expression that are affected (up, down, or unchanged) in Δ*elav*Δ*fne* mutants. (*F*) Heat map representing circRNA expression in neurons (differential expression in neurons compared with nonneurons) and in Δ*elav*Δ*fne* (differential expression in mutant compared with wild-type neurons). CircRNAs are grouped according to their expression in cell populations (neuronal, nonneuronal, and other). Only circRNAs significantly affected in Δ*elav*Δ*fne* are represented. (*G*) Differentially expressed circRNAs upon ELAV expression in S2 cells compared with an empty vector control. Significantly (*P* < 0.1) upregulated (log_2_FC ≥ 1; purple) and downregulated (log_2_FC ≤ −1; brown) circRNAs are highlighted. Dots are jittered to reduce overlap.

The RBP ELAV is a key regulator of neuron-specific alternative RNA processing across neuronal cell types, including the generation of neuron-specific (linear) splice isoforms (Carrasco et al. 2020, 2022; Wei et al. 2020; Lee et al. 2021; Torres-Méndez et al. 2022). To test a role for ELAV in circRNA expression, we compared the circular transcriptome of wild-type flow-sorted neurons with those of Δ*elav* and Δ*elav*Δ*fne* neurons. We found a marked and global decrease in neuronal circRNA expression in the absence of ELAV proteins, with >75% of neuronal circRNAs significantly downregulated (Fig. 2A,B). CircRNA depletion was more pronounced in Δ*elav*Δ*fne* double mutants (Fig. 2C,D; Supplemental Fig. S3A,B), showing that FNE rescues circRNA-related functions of ELAV like it does alternative linear RNA processing (Carrasco et al. 2020; Wei et al. 2020; Lee et al. 2021). In contrast, few neuronal circRNAs were affected in adult brains of Δ*fne*Δ*rbp9* mutant flies (Supplemental Fig. S3C), suggesting that the two cytoplasmic members of the ELAV protein family play only a minor role in the expression of neuronal circRNAs.

Clustering of circRNA expression in Δ*elav*Δ*fne* neurons identified a distinct pattern: Neuronal circRNAs were globally downregulated in Δ*elav*Δ*fne* mutants, whereas nonneuronal or “other” circRNAs were upregulated (Fig. 2D–F), showing that ELAV drives the neuronal identity (“neuronality”) of the circRNA landscape. Additionally, we noted the appearance of circRNAs in Δ*elav*Δ*fne* neurons not detected in any other cell population, “ectopic circRNAs,” most of which were undetectable (BSJ < 1) even in RNaseR-treated wild-type tissues (Supplemental Fig. S3D), implying that ectopic and upregulated circRNAs could represent an unspecific side effect of ELAV loss on splicing. To validate that neuronal circRNA expression changes are not caused by changes in gene expression, we assessed changes in gene expression within circRNA host genes as well as ratios between BSJs and forward-splicing junctions (FSJs). As expected, we observed higher BSJ/FSJ ratios for neuronal circRNAs in neurons compared with other cells; ratios decreased for neuronal, but not other, circRNAs in Δ*elav*Δ*fne* neurons (Supplemental Fig. S3E). In addition, most host genes of neuronal circRNAs were not significantly differentially expressed in Δ*elav*Δ*fne* neurons, with few cognate linear transcripts downregulated (*n* = 27) or upregulated (*n* = 16) (Supplemental Fig. S3F). These data are consistent with a model in which ELAV supports back-splicing at the expense of forward-splicing.

Next, we expressed ELAV in *Drosophila* S2 cells, which are macrophage-like with very low natural ELAV expression. Ectopic ELAV caused an upregulation of many circRNAs, with very few downregulated (Fig. 2G). Among 152 upregulated circRNAs, only three were neuronal (Supplemental Fig. S3G). This indicates that in addition to ELAV, the expression of neuronal circRNAs requires other factors expressed in neurons but not in S2 cells and that ELAV constitutes a general, positive regulator of circRNA expression. In summary, our data provide strong evidence that the pan-neuronal expression of ELAV protein is the main cause for the high circRNA diversity and abundance in neural tissues.

### ELAV binds to nascent RNAs but not mature circles

**Figure 3.**
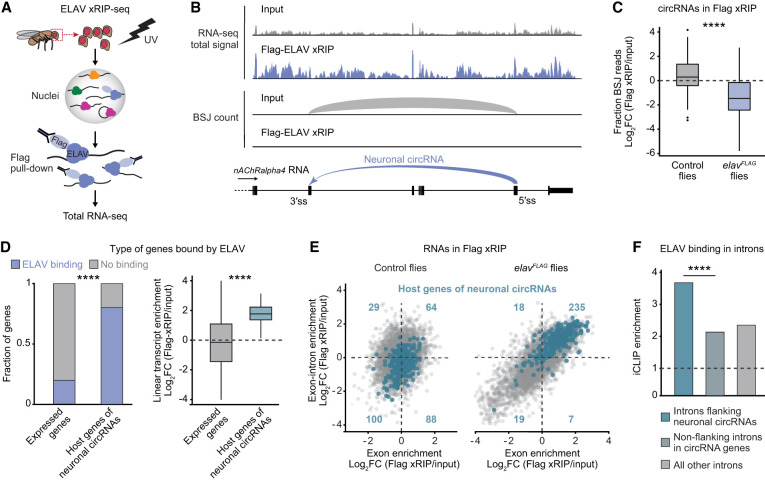
ELAV binds to pre-mRNA of neuronal circRNAs host genes. See also Supplemental Figure S4 and Supplemental Table S2. (*A*) xRIP-seq workflow. UV-cross-linked heads from adult flies underwent nuclear fractionation followed by isolation of protein–RNA complexes and RNA sequencing. In the shown example, FLAG-tagged ELAV was captured using anti-FLAG beads in flies of the genotype *elav*^*FLAG*^ or *w*^*1118*^ (control). (*B*) Total RNA-seq signal tracks and representation of the BSJ count of a portion of the gene *nAChRalpha4* in FLAG-ELAV xRIP compared with input. The arrow in the gene model indicates the back-splicing event that produces the neuronal circRNA. (*C*) ELAV binding to circRNAs in *elav*^*FLAG*^ and *w*^*1118*^ (control) flies, calculated as the ratio of circular to linear transcript expression in FLAG-ELAV xRIP compared with input. (****) *P* < 0.0001 (one-tailed Welch's *t*-test). (*D*, *left*) Proportion of genes that displayed significant enrichment in FLAG-ELAV xRIP-seq compared with input (log_2_FC > 1 and *P* < 0.01) in host genes of neuronal circRNAs and in all other genes (expressed in input sample). (****) *P* < 0.0001 (Pearson's χ^2^ test). (*Right*) Differential linear transcript expression in FLAG-ELAV xRIP compared with input for the same gene groups. (****) *P* < 0.0001 (one-tailed Welch's *t*-test). (*E*) Exon–intron split analysis of FLAG-ELAV xRIP compared with input. Each dot represents one gene. For each gene, ELAV enrichment at exon–intron boundaries (pre-mRNA) is shown as a function of ELAV enrichment in exons (total transcript). Host genes of neuronal circRNAs are highlighted in blue. Total transcript enrichment and pre-mRNA enrichment were calculated from exon reads and from reads overlapping exon–intron boundaries, respectively. (*F*) Enrichment of ELAV iCLIP signal in introns. Introns immediately upstream of or downstream from neuronal circRNA back-splice sites (flanking neuronal circRNAs) are compared for enrichment against other introns of the same gene (nonflanking) and introns of genes that do not host a neuronal circRNA (all other). (****) *P* < 2.2 × 10^−16^ (Pearson's χ^2^ test).

To test whether ELAV directly binds neuronal circRNAs in vivo, we performed ELAV RNA immunoprecipitation with UV-cross-linking followed by RNA sequencing (xRIP-seq) in extracts from adult head tissue (Fig. 3A). We ensured identification of high-confidence targets by using a polyclonal antibody directed against native ELAV (Carrasco et al. 2020) and, in an independent experiment, an anti-FLAG antibody in head extracts of flies in which the endogenous *elav* gene was N-terminally FLAG-tagged (*elav*^*FLAG*^ flies). Control xRIP-seq samples were obtained from head extracts of untagged flies treated with the anti-FLAG antibody. As expected, xRIP-seq recovered almost all transcripts previously identified as ELAV functional targets for AS or APA (Supplemental Fig. S4A; Carrasco et al. 2020, 2022; Wei et al. 2020; Lee et al. 2021). However, we found that ELAV does not bind circRNAs: BSJs were strongly underrepresented in xRIP samples compared with input (Fig. 3B,C; Supplemental Fig. S4B–D). In contrast, xRIP samples were highly enriched in reads originating from genes that host neuronal circRNAs (Fig. 3B,D; Supplemental Table S2). To discriminate between pre-mRNA and mRNA binding, we performed an analysis based on eisaR (Gaidatzis et al. 2015): We compared the enrichment of signals from exon–intron boundaries (pre-mRNA) against that from exonic sequences (pre-mRNA and mRNA) for each protein-coding gene. Remarkably, 90% of genes from which neuronal circRNAs originate were enriched at the pre-mRNA level (Fig. 3E; Supplemental Fig. S4E,F). From these data, we conclude that ELAV binds to pre-mRNAs of select genes to promote neuronal circRNA biogenesis. The absence of ELAV binding to closed circles could mean that ELAV binds to intronic sequences that will later be spliced out of the pre-mRNA. Indeed, analysis of ELAV iCLIP data from adult fly heads (Carrasco et al. 2020) revealed a significant ELAV binding enrichment in introns that flank neuronal circRNAs (Fig. 3F). Together, our data demonstrate that ELAV globally mediates neuronal circRNA expression through binding the pre-mRNA of circRNA host genes.

### ELAV binds to RCMs to inhibit forward-splicing and promote back-splicing

**Figure 4.**
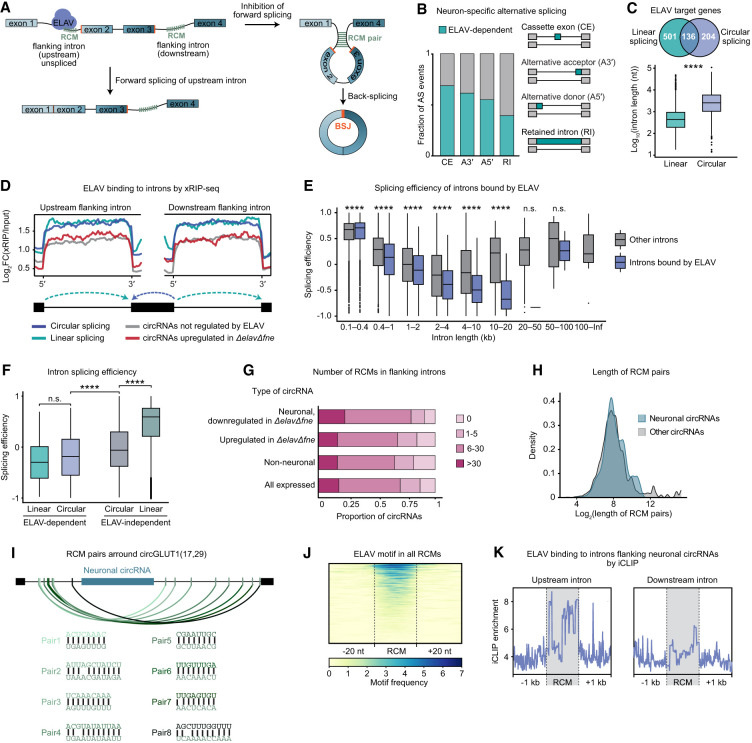
ELAV binds to RCMs to inhibit forward-splicing and promote back-splicing. See also Supplemental Figure S5 and Supplemental Tables S3 and S4. (*A*) Model of ELAV-mediated circRNA biogenesis: ELAV binds to reverse complementary match (RCM) sequences in introns flanking the circRNA back-splice junction (BSJ) site and inhibits splicing efficiency, resulting in secondary structures that promote neuronal circRNA expression. (*B*) Proportion of neuronal alternative splicing events (differential splicing events in neurons compared with nonneurons) that are ELAV-dependent (differential in Δ*elav*Δ*fne* compared with wild-type neurons) for the indicated types of splicing. (*C*, *top*) Venn diagram showing the number of genes that undergo ELAV-dependent alternative splicing (linear) and genes that host an ELAV-dependent circRNA (circular). (*Bottom*) Box plot representing the length of the flanking introns for each type of splicing event. (****) *P* < 0.0001 (one-tailed Welch's *t*-test). (*D*) Gene metaplot representing ELAV xRIP-seq enrichment in flanking introns of the indicated types of ELAV-regulated splicing events. Forward and reverse arrows in the gene model represent linear and back-splicing events, respectively. (*E*) Splicing efficiency of introns bound by ELAV (by xRIP-seq) compared with other introns in function of intron length. Introns were grouped in bins by length. (****) *P* < 0.0001 (one-tailed Welch's *t*-test), (n.s.) not significant. (*F*) Splicing efficiency of introns undergoing linear splicing or back-splicing, comparing ELAV-regulated AS and circRNA formation with ELAV-independent splicing events. (****) *P* < 0.0001 (one-tailed Welch's *t*-test). (*G*) For each type of circRNA, the proportion of circRNAs whose flanking introns contain reverse complementary match (RCM) sequences is shown. Number ranges indicate RCMs per intron. (*H*) RCM density in function of RCM pairing length in introns flanking the indicated types of circRNAs. RCM pairs flanking the same circRNAs were grouped as one pair. (*I*) Representation of a portion of the circRNA-hosting gene *Glut1*, showing RCM pairs and their sequences. The back-splicing event that produces the neuronal circRNA *circGlut1(17,29)* is indicated. Introns flanking neuronal circRNAs are zoomed to show specific pairing. (*J*) Heat map showing the number and distribution of ELAV motifs in RCM regions of introns flanking neuronal circRNAs. RCMs of introns that contain at least one ELAV motif (independently of the motif's position within the intron) are displayed. (*K*) Profile plot of ELAV iCLIP signal at RCMs and in 1 kb surrounding in introns flanking (upstream and downstream) neuronal circRNAs.

We hypothesize that in neurons, ELAV binds to flanking introns of neuronal circRNAs in the nascent transcripts to promote intron pairing and back-splicing (Fig. 4A). To assess whether the role of ELAV in circRNA biogenesis is linked to its established function in regulating neuronal AS and/or APA, we quantified (linear) splice junction counts in wild-type and Δ*elav*Δ*fne* neurons (Supplemental Table S3), thereby identifying neural-specific splice events. We found that the majority of (linear) neuronal splicing events are ELAV-dependent (Fig. 4B; Supplemental Table S3), consistent with prior studies demonstrating the impact of ELAV proteins in the establishment of neuronal mRNA signatures (Carrasco et al. 2020; Lee et al. 2021) and also suggesting that ELAV's role in AS is more extensive than previously recognized. Notably, most (60%) ELAV-dependent circRNAs are produced from genes with no measurable linear AS, indicating that ELAV can independently regulate linear and circular splicing (Fig. 4C). However, many host genes of ELAV-dependent circRNAs display ELAV-dependent AS (40%), APA (21%), or both (13%) (Supplemental Fig. S5A; Supplemental Table S4), expanding previous observations that ELAV-mediated alternative RNA processing events co-occur (Zhang et al. 2019; Carrasco et al. 2022; Zhang et al. 2023) and may be coregulated.

CircRNAs are often flanked by long introns (Jeck et al. 2013; Westholm et al. 2014); comparing linear and circular splicing targets of ELAV, we noted a nearly 10-fold difference in median intron length (Fig. 4C). We measured ELAV xRIP signal in introns of genes undergoing either neuronal AS or neuronal back-splicing and found comparable and high enrichment of ELAV binding in both scenarios (Fig. 4D). These data show that the ELAV-dependent generation of neuronal circRNAs constitutes a regulated process that likely evolved to benefit neuron development and functionality. In contrast, ectopic or ELAV-independent circRNAs did not display features of specific regulation (Fig. 4D; see also Figs. 1D, 2D–F; Supplemental Fig. S3D) and may constitute nonfunctional or even deleterious side products of linear splicing, as has been proposed to be the case for many mammalian circRNAs (Xu and Zhang 2021).

One possible mechanism through which ELAV binding promotes back-splicing is by inhibiting linear splicing of long introns that flank circRNAs. We evaluated cotranscriptional splicing efficiency using nascent RNA-seq data from *Drosophila* heads, comparing signal in unspliced introns with that in their respective downstream exons (Supplemental Fig. S5B; Khodor et al. 2012). Consistent with findings in *Drosophila* S2 cells (Pai et al. 2017), splicing efficiencies generally decreased with intron length (up until 10 kb) (Fig. 4E). Accordingly, the typically long introns flanking circRNAs exhibited significantly lower splicing efficiencies than non-circRNA-associated introns of the same gene (Supplemental Fig. S5C). Interestingly, ELAV-dependent introns as well as introns bound by ELAV are spliced significantly less efficiently compared with other introns, independently of intron length, whether in the context of AS or circRNA formation (Fig. 4E,F). These observations suggest that ELAV binding reduces splicing efficiency, thereby increasing the window of opportunity for AS and back-splicing.

We also hypothesized that ELAV binding may facilitate the formation of secondary structures that promote intron pairing at reverse complementary match (RCM) sequences (Fig. 4A). RCMs constitute hallmarks of interintronic interaction and can predict circRNA formation (Liang and Wilusz 2014; Zhang et al. 2014; Ivanov et al. 2015), though not consistently. In *Drosophila*, repeat elements resembling RCMs were shown to drive circularization (Kramer et al. 2015; Pamudurti et al. 2022), but several studies have argued against a systematic role for RCMs in circRNA formation (Ashwal-Fluss et al. 2014; Westholm et al. 2014). We found that RCMs in introns flanking circRNAs significantly overlapped (12%) (Supplemental Fig. S5D) with repeat regions in the *Drosophila* genome, with a particularly high enrichment for Helitron transposable elements (Supplemental Fig. S5E). We identified RCMs in flanking introns of most circRNAs, with a particular prevalence in ELAV-dependent circRNAs (Fig. 4G–I). Compared with other sequences within the same intron, RCM regions bracketing neuronal circRNAs displayed similar conservation scores but a particularly low CG content (Supplemental Fig. S5F,G). These RCMs were also highly enriched in ELAV binding motifs (Fig. 4J). An orthogonal motif search comparing RCMs with the remainder of the sequence of circRNA-flanking introns also yielded ELAV as the top predicted binder (Supplemental Fig. S5H,I). ELAV iCLIP from brain tissue showed significant local binding of ELAV at RCMs flanking neuronal circRNAs, with signal particularly strong in the upstream intron; i.e., the intron transcribed first (Fig. 4K). Together, these results indicate that ELAV binds to RCMs in introns upstream of neuronal circRNAs and inhibits linear splicing, thereby preserving upstream RCMs for base pairing with downstream RCMs and promoting back-splicing (Fig. 4A).

### Deletion of RCM regions abrogates neuronal circRNA expression

**Figure 5.**
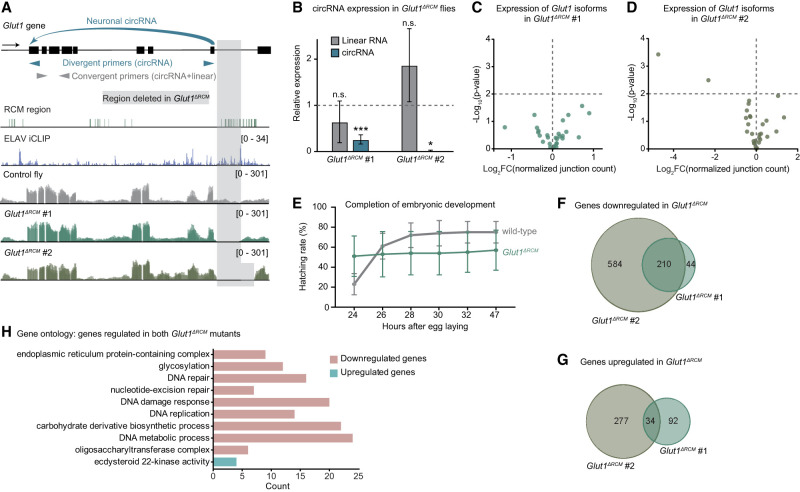
Deletion of RCM regions abrogates neuronal circRNA expression in the *Glut1*^Δ*RCM*^ mutant. See also Supplemental Table S5. (*A*) Representation of a portion of the circRNA-hosting *Glut1* gene region, showing RCMs, ELAV iCLIP signal, mRNA-seq tracks from *Glut1*^Δ*RCM*^ mutant flies and controls, and the region deleted in *Glut1*^Δ*RCM*^ mutant flies. The back-splicing event that produces the neuronal circRNA *circGlut1(17,29)* is indicated. Small arrows represent primer pairs used in the RT-qPCR shown in *B*. Divergent primers are specific to the circRNA, whereas convergent primers detect both circular and linear transcripts. (*B*) RT-qPCR quantification of circular and linear *Glut1* transcripts in *Glut1*^Δ*RCM*^ mutant embryos (at 18–20 h; two independent mutants). RNA levels were normalized to *RpL32 (rp49)* mRNA, and levels in control embryos were set to the value 1 (dotted line). Error bars represent mean ± SD of four biological replicates for each genotype. (n.s.) Not significant, (*) *P* < 0.05, (***) *P* < 0.001 (unpaired Student's *t*-test comparing mutant with control). Control embryos are heterozygous progeny of mutant (homozygous) embryos. (*C*,*D*) Volcano plot showing differentially expressed *Glut1* mRNA isoforms in *Glut1*^Δ*RCM*^ mutant embryos (at 18–20 h; two independent mutants). The *P* = 0.01 cutoff is shown as a horizontal line. (*E*) Embryonic viability in *Glut1*^Δ*RCM*^, measured as the proportion of embryos that hatched into larval stage 1. (Wild-type) *w*^*1118*^ flies. Error bars indicate mean ± SD of 10 biological replicates. (*F*,*G*) Venn diagrams showing the number of downregulated (log_2_FC < −1 and *P*_adj_ < 0.05; *F*) and upregulated (log_2_FC > 1 and *P*_adj_ < 0.05; *G*) genes identified in *Glut1*^Δ*RCM*^ mutants #1 and #2. Data are from 18–20 h embryos. (*H*) Gene ontology classification (GO terms) of genes regulated in both *Glut1*^Δ*RCM*^ mutant #1 and #2, ranked by significance.

To functionally validate that circRNA formation is mediated by ELAV-bound RCMs in vivo, we generated the fly mutant *Glut1*^Δ*RCM*^, in which RCMs and ELAV binding sites downstream from *circGlut1(17,29)*, a neuronal circRNA, were deleted by CRISPR/Cas9-mediated gene editing. The gene *Glut1* was selected because it expresses a well-defined neuronal circRNA at relatively high levels as well as ELAV iCLIP signal in RCMs of both flanking introns. We generated two independent mutants differing by the size of the genomic deletion (*Glut1*^Δ*RCM*^ #1 and #2) (Fig. 5A). In both mutants, expression of the circular, but not the linear, *Glut1* transcript was severely reduced (Fig. 5B). We performed mRNA-seq on embryos of both mutants to assess the effect of the deletion on linear splicing and isoform expression. Interestingly, alternative splicing was not affected in mutant #1, whereas the larger deletion in mutant #2 caused not only circRNA depletion but also disruption of individual mRNA variants (Fig. 5A,C,D; Supplemental Table S5). *Glut1*^Δ*RCM*^ mutant #1 flies showed a mildly reduced adult viability, with close to 50% failing to complete embryonic development and hatch into larvae (Fig. 5E). Surmising that these defects are caused by direct or indirect effects of *circGLUT1(17,29)* depletion, we identified ∼200 genes downregulated in both *Glut1*^Δ*RCM*^ mutants (Fig. 5F,G). A gene ontology analysis on this gene set revealed an enrichment in genes encoding proteins involved in protein glycosylation and DNA repair (Fig. 5H), cell processes previously shown to be associated with circRNA biology in *Drosophila* (Weigelt et al. 2020) and in human cells (Conn et al. 2023; Li et al. 2023; Shu et al. 2023). These phenotypes indicate a mild and pleiotropic role for the neuronal *Glut1* circRNA in animal physiology.

## Discussion

In this study, we demonstrated that the pan-neuronal protein ELAV is the global mediator of neuronal circRNA synthesis; ELAV's tissue-specific expression underlies the extraordinary abundance of circRNAs in the nervous system. Mechanistically, ELAV binds to the pre-mRNA of genes that produce neuronal circRNAs, specifically targeting RCM sequences in BSJ-flanking introns. Long introns are associated with circRNA formation in flies (Westholm et al. 2014; this study) and mammals (Jeck et al. 2013; Salzman et al. 2013; Zhang et al. 2014) as well as with low splicing efficiencies (Wilusz 2017; Zeng et al. 2022), which supports the notion that many circRNAs constitute functionally neutral or deleterious side products of splicing. Our data indicate that most neuronal circRNAs do not belong to that category: ELAV-dependent circRNA formation occurs in a regulated and highly specific fashion, with reduced splicing efficiencies in ELAV-bound introns. In our model, ELAV binding to the RCM in the upstream flanking intron represents the crucial step, ensuring retention of that intron and its RCM while the downstream intron is being transcribed, which can take several minutes in long neuronal genes. It is still not clear how ELAV achieves the targeting specificity required to induce circularization at distinct neuronal BSJs. The ELAV binding motif is found broadly distributed across the genome and cannot by itself account for the observed positional enrichment at RCMs. The prevalence of AU-rich regions in ELAV-bound RCMs that flank neuronal circRNAs is reminiscent of the high frequency of Alu elements in the introns of primates, where Alu pairing regulates linear and circular splicing (Sorek et al. 2002; Zhang et al. 2014; Aktaş et al. 2017; Liang et al. 2023). Along species evolution, the higher number of complementary elements in longer introns (Zhang et al. 2014) may contribute to the increased complexity of circRNA expression (Dong et al. 2017). Even within the nervous system, circRNAs display cell type-specific expression patterns (Dube et al. 2019; Ji et al. 2019; Wu et al. 2020); it is possible that the combination of transcription and RNA processing factor specificities selects and modulates transcript circularization in distinct neurons, perhaps even in distinct synapses.

In fly embryos, at least 75% of neuronal circRNAs depend on ELAV/FNE. The accumulation of circRNAs in the brain is a molecular landmark across animals (Westholm et al. 2014; Rybak-Wolf et al. 2015; Liu and Chen 2022). Considering the similarity of neuronal ELAV-like (nELAV) proteins between flies and humans in terms of sequence and expression patterns (Wei and Lai 2022; Hilgers 2023; Mulligan and Bicknell 2023), conserved mechanisms may be at play to regulate intron pairing and neuronal circRNA biogenesis. Moreover, the predominantly cytoplasmically localized human nELAV protein HuD binds to a quarter of all brain-expressed circRNAs as well as many host transcripts (Dell'Orco et al. 2020); in context with our findings, this raises the hypothesis that in evolutionarily distant species, nuclear nELAV proteins (such as ELAV) mediate circRNA biogenesis and that binding of mature circRNAs by cytoplasmic nELAVs (such as HuD) regulates their local expression and function. The exquisite coregulation of neuronal circRNAs at the levels of biosynthesis (Knupp et al. 2021; this study), subcellular localization, and response to extrinsic signals (Rybak-Wolf et al. 2015; You et al. 2015; Cao et al. 2024) could indicate that neuron-specific, nELAV-dependent circRNAs may function in concert; for example, by binding a common set of RBPs. Our finding that BSJ regions of neuronal circRNAs are enriched for binding motifs for neuronal RBPs is consistent with this possibility. RBPs often localize in synapses, where they regulate the local translation and transport of neuronal transcripts (Prashad and Gopal 2021; Sun and Schuman 2023). With their long half-life, unique RBP binding sites, and versatile functionalities, circRNAs possess great potential for modulating gene expression in a cell-specific and even synapse-specific manner.

## Materials and methods

Further information about resources and reagents is available on request.

### Data and code availability

All sequencing data generated during this study can be accessed at NCBI Gene Expression Omnibus under the accession number GSE269179. The code used in this study to generate all figures has been deposited at https://github.com/hilgers-lab/circles2024 and https://github.com/hilgers-lab/SpliceFlow.

### Experimental model and genome editing

In this study, all experiments used *Drosophila melanogaster* embryos. The ages of the embryos are specified in hours after egg laying (AEL) when maintained at 25°C. Control flies (*w*^*1118*^) and GFP-marked balancer chromosomes were sourced from the Bloomington Stock Center (lines 5905, 4559, and 6662). Null alleles for *fne*, denoted as Δ*fne*, were acquired from M. Soller (Zaharieva et al. 2015); Δ*elav* and *fne*^*FLAG*^ alleles were from Carrasco et al. (2020). *elav*^*FLAG*^*elav*^*RBD*^ and *Glut1*^Δ*RCM*^ fly lines were generated through CRISPR/Cas9 genome editing, adhering to the methods outlined by Port and Bullock (2016). Embryo injections were performed by BestGene, Inc. *elav*^*FLAG*^ expressing an endogenously, N-terminally FLAG-HA-tagged ELAV protein was generated using a guide RNA (GTCTACTCCGCCGCCAGCTC) targeting *elav* and a plasmid comprising 1406 nt upstream of the tag insertion site, the FLAG-HA sequence, and 1509 nt downstream from the insertion site (sequence shown in Supplemental Table S6). To create *elav*^*RBD*^, a total of 10 single-nucleotide mutations in all six RNP motifs of the three ELAV RRMs, previously described to eliminate ELAV RNA binding (Lisbin et al. 2000), was introduced by injecting the *elav*^*FLAG*^ line with a plasmid containing two guide RNAs (AACCACAGCAGGCGCAGCCC and GTCTACTCCGCCGCCAGCTC) and a 1257 nt gBlock (Integrated DNA Technologies) as a homology donor (sequence shown in Supplemental Table S6). The resulting amino acid mutations were as follows: for RRM1 and RNP1, Y205A and F207D; for RRM1 and RNP2, I152A and N154A; for RRM2 and RNP1, F294A; for RRM2 and RNP2, Y251A; for RRM3 and RNP1, Y445A and F447D; and for RRM3 and RNP2, F405A and Y407A. *Glut1*^Δ*RCM*^ flies were generated by injecting a plasmid containing two guide RNAs (ATGAGGATGCGGAGGATTC and ACTTGATTATTACAATGCA) targeting the RCM region in the *Glut1* gene. The two resulting mutants, #1 and #2, carried genomic deletions of differing lengths.

### Quantification of embryonic viability

Embryonic viability was calculated as the fraction of embryos that successfully completed embryogenesis and hatched into first instar larvae. Starting at 22–24 h after egg laying (AEL), the number of hatched embryos was recorded every 2 h for 12 h and one last time at 45–47 h AEL.

### Genetic strategy to sort wild-type and Δ*elav* mutant neurons (14–16 h data set)

We used the progeny of Δ*elav,fne^FLAG^/FM7* and FM7/Y flies that contained the *elav*-null allele Δ*elav* (Carrasco et al. 2020) recombined with endogenously V5-flagged *fne (fne*^*FLAG*^) (Grzejda et al. 2022). FLAG was used as a marker of neuronal cells, and ELAV protein was used as a marker of wild-type neurons, which allowed us to distinguish Δ*elav* mutant neurons (FLAG^+^, ELAV^−^), wild-type neurons (FLAG^+^, ELAV^+^), and nonneuronal cells (FLAG^−^, ELAV^−^).

### Genetic strategy to sort wild-type and Δ*elav*Δ*fne* mutant neurons (18–20 h data set)

Flies described as Δ*elav*Δ*fne* flies were progeny of *elav^RBD^,*Δ*fne*/FM7 females crossed with FM7/Y males. As a result, the RNA-binding dead ELAV protein could be discriminated from the wild-type ELAV by FLAG and never coexpressed with FNE. In heterozygous flies, the *elav*^*RBD*^ allele was robustly suppressed (through a mechanism we have not characterized), leading to the absence of detectable *elav*^*RBD*^ protein (Supplemental Fig. S1A). This suppression allowed for the positive identification of neuronal populations that lacked the ELAV^RBD^ protein using FLAG as a marker. Wild-type neurons (FLAG^−^ and ELAV^+^) were distinguished from mutant neurons (*elav^RBD^,*Δ*fne*: FLAG^+^, ELAV^+^), and nonneuronal cells were identified as FLAG^−^, ELAV^−^.

### RNaseR treatment

For head samples, 3 day old *w*^*1118*^ flies were collected, flash-frozen in liquid nitrogen, and decapitated by shaking, and heads were collected manually with a thin brush. For embryo samples, eggs from *w*^*1118*^ flies were collected for 2 h on agar plates and aged for 14 h (14–16 h AEL embryos) at 25°C. In both cases, samples were homogenized in QIAzol lysis reagent (Qiagen 79306) for RNA extraction. Two micrograms of RNA per sample was treated with 10 U of RNaseR (Applied Biological Materials E049) in 20 µL for 20 min at 37°C. For nontreated control samples, the enzyme was omitted. After addition of spike-in mouse embryonic stem cell RNA at 0.33 ng/µL final concentration, RNA was extracted with TRIzol LS (Ambion 10296028) and glycogen (Invitrogen AM9515) according to the manufacturer's protocol. RNAs were reverse-transcribed using Maxima RT (Thermo Scientific EP0741) with random hexamers (Jena Bioscience 94824), according to the manufacturer's protocol.

### RNA sequencing library preparation

RNA integrity was analyzed using a 2100 Bioanalyzer (Agilent Technologies). Libraries for total RNA-seq were prepared with 100 ng of total RNA using TruSeq stranded total RNA library preparation (Gold, Illumina 20020599) according to the manufacturer's instructions. Paired-end sequencing was performed using the NovaSeq 6000 platform (Illumina) and 101 bp reads. Libraries for mRNA-seq were prepared using Illumina stranded mRNA preparation (Illumina 20040534), and paired-end sequencing was performed using the NovaSeq 6000 platform (Illumina) and 150 bp reads.

### Fluorescence-activated cell sorting in *Drosophila* embryos

*Drosophila* embryos were collected at the appropriate times after egg laying. Sorting of embryo populations was done based on McCorkindale et al. (2019) with modifications. All steps until FACS were carried on at 4°C, and all washes used cold, RNase-free PBS (Alfa Aesar J62851) with RNase inhibitor (1:250; RiboLock, Thermo Fisher) and 1000*g* for 2 min to preserve RNAs from degradation. Embryos were dounced in RNase-free PBS with RNase inhibitor (1:250), and cell debris were filtered out by filtration through a 40 µm strainer at 400*g*. Cells were washed twice and stained with Zombie Aqua (BioLegend 423101) for live/dead separation for 15 min in the dark in PBS with 1:100 RNase inhibitor. Cells were washed twice and then fixed with 4% formaldehyde (Thermo Fisher Scientific 28908) in PBS for 15 min in the dark. Fixation was stopped with incubation for 3 min with a quenching buffer (750 mM Tris-HCl at pH 7.5 in DEPC-PBS, 1:100 RNase inhibitor), and cells were washed twice again. Cells were incubated with the primary antibodies polyclonal rabbit anti-ELAV (1:500; generated by Carrasco et al. [2020]) and mouse anti-FLAG M2 (1:500; Sigma F1804) for 45 min at 4°C in the dark with agitation in staining buffer (1% [w/v] BSA[Sigma 6917], 0.1% saponin, 1:200 RiboLock). Cells were washed twice in 0.2% BSA, 0.1% saponin in PBS, and 1:100 RiboLock and incubated with fluorescently conjugated antibodies at 1:500 for 14–16 h embryos or at 1:1000 for 18–20 h embryos for 45 min at 4°C in the dark with agitation in staining buffer. Sorting used 488 antimouse (Abcam) and 555 antirabbit (Invitrogen) in Δ*elav* embryos and 488 antirabbit (Life Technologies) and 555 antimouse (Abcam) in Δ*elav*Δ*fne* embryos. Cells were then washed twice, resuspended in sorting buffer (0.5% BSA, 2 mM EDTA, 1:500 RiboLock), filtered through a 40 µm nylon mesh, sorted using a FACSymphony (Becton Dickinson), and collected in cold sorting buffer. Cells were pelleted, and RNA–protein complexes were reverse-cross-linked with proteinase K (Ambion AM2546) in 10 mM Tris (pH 8), 10 mM NaCl, 1 mM EDTA, 1:100 proteinase K, and 1:100 RiboLock for 20 min at 50°C. RNA was extracted in 3:1 TRIzol LS (Ambion) according to the manufacturer's protocol.

### Cell transfection

S2 cells were transfected in 6 well plates using Effectene (Qiagen) with 300 ng of the *λ*N expression plasmid (*λ*N empty plasmid or *λ*N-ELAV from Hilgers et al. 2012). Transfections were conducted in three replicates. Cells were harvested 48 h after transfection by centrifugation and lysed in QIAzol lysis reagent (Qiagen 79306) for RNA extraction.

### CircRNA validation by qPCR

Δ*elav*Δ*fne* and *Glut1*^Δ*RCM*^ flies were raised in heterozygosis with GFP-marked balancer chromosomes. Embryos were dechorionated following standard procedures and placed on a plate containing halocarbon oil. Mutant embryos were selected according to embryonic age and morphology and against GFP signal. For each replicate, 300–500 ng of total RNA was used. RNA was reverse-transcribed using Maxima RT (Thermo Scientific EP0741) with random hexamers (Jena Bioscience 94824) according to the manufacturer's protocol. RT-qPCR was performed in a LightCycler 480 II instrument using FastStart SYBR Green Master (Roche). Divergent primers were designed to amplify only circular RNAs from the target genes. RT-qPCR primer sequences are listed in Supplemental Table S6.

### Cross-linking RNA immunopurification and sequencing (xRIP-seq)

*Drosophila* heads were separated from bodies by shaking in liquid nitrogen and ground into powder in liquid nitrogen. For one sample, 100 mg of head powder was subjected to 254 nm UV-cross-linking (UV-C;) through six rounds at 300 mJ/cm² in a BLX-312 cross-linker (Bio-Link). All subsequent steps were performed at 4°C. Nuclear extracts were obtained by homogenizing the UV-cross-linked head powder in 1 mL of homogenization buffer (0.2% Triton X, 10% sucrose, 0.5 mM DTT, double the standard concentration of protein inhibitors [Roche 11873580001], 1:500 RiboLock RNase inhibitor [Thermo Fisher Scientific EO0384]), using 10 strokes with a loose pestle. For each replicate, three samples (3 mL of homogenate) were pooled, and homogenate was filtered using a 40 µm strainer and centrifuged at 200*g* for 3 min. Supernatants were transferred to 15 mL Protein LoBind (Eppendorf 0030122216) tubes and centrifuged at 800*g* for 10 min to pellet nuclei. Pellets were resuspended and centrifuged at 600*g* for 10 min to pellet nuclei in 6 mL of homogenization buffer excluding Triton X and centrifuged at 800*g* for 10 min to pellet nuclei. This pellet was then resuspended in lysis buffer (50 mM Tris at pH 7, 500 mM LiCl, 10 mM EDTA, 5 mM DTT, 2% lithium dodecyl sulfate [LDS], 0.5% SDS, 0.5% sodium deoxycholate) and rotated for 10 min. The lysate was centrifuged at 15,000*g* for 10 min, and the supernatant was transferred to a new tube. After another centrifugation under the same conditions, ionic detergents were neutralized with 1% NP-40. For the FLAG-ELAV experiment, 750 µL of the clarified lysate (referred to as “input”) was incubated with 40 µL of anti-FLAG M2 magnetic beads (Invitrogen M8823) for 1.5 h. The beads were rinsed with lysis buffer (0.1% SDS, 0.1% Na-deoxycholate), washed three times for 5 min with lysis buffer (0.1% SDS, 0.1% Na-deoxycholate, 1% Triton X-100), and washed twice with lithium chloride buffer (350 mM LiCl, 50 mM HEPES-KOH at pH 7.5, 1 mM EDTA, 1% NP-40, 0.7% Na-deoxycholate) for 5 min. Protein–RNA complexes were eluted with 120 µL of elution buffer (10 mM Tris-HCl at pH 7.4, 150 mM NaCl, 0.1% Triton X-100, without protease inhibitors) containing 0.2 mg/mL FLAG peptide (Sigma-Aldrich F3290) for 1 h. The eluates and corresponding inputs underwent treatment with proteinase K (Ambion AM2546) at 1100 rpm for 30 min at 50°C. For the endogenous ELAV IP experiment, input was incubated with 40 µL of the conjugated Dynabeads protein A–antibody complex with rabbit anti-ELAV antibody (generated by Carrasco et al. 2020) for 1 h. Protein–RNA complexes were eluted in 120 µL of 1× Proteinase K buffer (Tris-HCl at pH 7.5, NaCl EDTA, 6 µL of 10% SDS, 1.5 µL of 20 mg/mL Proteinase K, 1 µL of RiboLock). RNA was then purified using TRIzol LS reagent (Ambion 10296028) following the manufacturer's protocol. Equal volumes of total RNA from both input and IP samples were used to prepare libraries for total RNA-seq.

### RNA sequencing data processing

Sequencing data were processed using the RNA-seq module from snakePipes v2.4.3 (Bhardwaj et al. 2019), adding flags for ‐‐trim, -m “alignment-free,alignment.” Reads were mapped to the *D. melanogaster* reference genome (Ensembl assembly release dm6) and the transcriptome reference annotation release-96 using STAR (Dobin et al. 2013). Quality control of RNA-seq reads was done using FASTQC (http://www.bioinformatics.babraham.ac.uk). To compare gene expression estimates across cell populations, a variance-stabilizing transformation (VST) was applied using the DESeq2 (Love et al. 2014) function vst() on raw gene count data from the different samples. The transformed data were used to compute a PCA using the DESeq2 function plotPCA() with standard parameters. Differential gene expression analysis was performed using DESeq2 filtering for baseMean ≥ 4, log_2_FC > 1, and *P*_adj_ < 0.01 to determine neuronal enriched genes. To determine differentially expressed genes in *Glut1*^Δ*RCM*^ mutants, analysis was performed using log_2_FC > 1 or log_2_FC < −1 and *P*_adj_ < 0.05.

### Generation of the circRNA reference database

The circRNA reference database was constructed from a compilation of libraries from RNaseR-treated RNA samples (from heads and embryos) and from RNA samples obtained from flow-sorted embryonic cell populations. For each group, replicates were pooled. Libraries were aligned using the Burrows–Wheeler alignment tool with the parameter “-T 19” (Li and Durbin 2009), and circRNAs were identified using CIRI2 under default settings as recommended by the tool's developers (Gao et al. 2018), based on the dm6 genome and Ensembl 96 annotations. This process involved filtering and consolidating the identified circRNA entities. Filtering criteria included a minimum of five back-splice junctions (BSJs) per circRNA per cell population, with mutant-specific circRNAs identified by the presence of more than five BSJs across the combined single- and double-mutant libraries. This consolidated collection of circRNAs formed the reference database that was used in subsequent analyses. Relative circRNA expression levels compared with their cognate linear RNAs were calculated in CIRIquant. This was expressed as the junction ratio, which was the circular to linear ratio calculated by the formula 2 × bsj/(2 × bsj + fsj). To validate circRNAs identified from CIRI2, the same libraries were aligned again with STAR aligner (2.7.0) based on the dm6 genome and Ensembl 96 annotation from UCSC. CircRNAs were identified and counted using CircSplice (Feng et al. 2019) under default settings.

### Saturation analysis (subsampling)

Saturation analysis was performed by pooling all replicates from one sample group and randomly sampling different fractions from 1% to 100% from the raw read files using seqtkV1.2-r94 (Shen et al. 2016). Next, the CIRI2 (Gao et al. 2018) pipeline was applied to each of the sample fractions. Results were summarized as a fraction of recovered compared with the full set.

### Differential circRNA expression

We computed differential circRNA expression by executing the CIRIquant (Zhang et al. 2020) workflow and using the circRNA reference database. CIRIquant was run with circRNA in format “‐‐ciri ‐‐library-type 2” and using the script prepDE.py to handle replicates to compute the differential status for each circRNA. After the circRNA quantification with CIRIquant, circRNAs were classified based on their expression in a given embryonic cell population compared with another cell population. We constructed four groups: two for the neuronal comparison (neuronal and nonneuronal) and two for the mutant comparison (ELAV-downregulated and ELAV-upregulated). For classification, we required that a circRNA comply with edgeR (Robinson et al. 2010) parameters of logCPM > −5, *P* < 0.1, and |log_2_FC| > 0. Differential circRNA expression analysis was also performed from a circRNA count table generated using CircSplice with edgeR and the same settings as CIRI_DE.R script in CIRIquant. Code for the CircSplice analysis is available at https://github.com/hilgers-lab/circles2024/CircSplice.snakemake.

### Motif enrichment at BSJs

RBP enrichment in BSJs of neuronal circRNAs was performed by generating a sequence of 50 nt flanking the BSJ site (±25 nt) using the BSgenome.Dmelanogaster. UCSC.dm6 reference genome package in R. The FASTA files were submitted to the MEME suite server, and the AME program (McLeay and Bailey 2010) was used to calculate enrichment over the sequences. CentriMo (Bailey and Machanick 2012) was used for assessing positional enrichment. For the comparisons, circRNAs that were not enriched or depleted in neurons were used as control sequences.

### Identification of ELAV target genes by xRIP-seq

To identify ELAV target genes, we used the DESeq2 pipeline DESeq (Love et al. 2014) and assessed read counts in xRIP compared with input. Prior to applying DESeq2, we filtered for genes with >10 read counts across all samples and replicates. For xRIP-seq performed with the anti-ELAV antibody in wild-type fly heads, DESeq2 was executed with default parameters using a simplified design, “design = ∼type,” where “type” represents the comparison between input and IP. For xRIP-seq performed with the anti-FLAG antibody in *elav*^*FLAG*^ flies, we used a likelihood ratio test with the following parameters: “design = ∼type + condition + condition:type” and “DESeq(test = ‘LRT,’ reduced = ∼type + condition).” In this context, “condition” accounts for the confounding factor of nonspecific binding to the anti-FLAG antibody, which we controlled for by performing FLAG IP in untagged *w*^*1118*^ flies. ELAV target genes for each xRIP experiment were selected with the criteria *P*_adj_ < 0.05 and log_2_FC > 1.

### Exon–intron split analysis (EISA) on xRIP-seq data

We counted reads specifically spanning exon–exon junctions (EEJs) and exon–intron boundaries (EIBs). EIB reads were defined as reads that did not span exon–exon junctions and fully matched the reference genome, indicated by having only “M” (match or mismatch) in their CIGAR string from STAR mapping. These reads were assigned to regions that overlapped both exons and introns. EEJ and EIB reads were counted using featureCounts (Liao et al. 2014) with the parameters “featureCounts -p -B -C -t ‘gene’ -g ‘gene_id’ -f -s 2 –J.” EISA was performed using the R package eisaR with the following parameters from Gaidatzis et al. (2015): runEISA(modelSamples = FALSE, geneSelection = ‘filterByExpr,’ statFramework = ‘QLF,’ effects = ‘predFC,’ pscnt = 2, sizeFactor = ‘individual,’ recalcNormFactAfterFilt = TRUE, and recalcLibSizeAfterFilt = FALSE). In this analysis, read counts from EEJs were used for mRNA counts, and reads of EIBs were used for intron counts (pre-mRNA). Genes with <10 EEJ counts on average across replicates were discarded. Code for the xRIP-seq EISA is available at https://github.com/hilgers-lab/circles2024/eisaR.

### ELAV iCLIP data processing

Previously published ELAV iCLIP data were processed as described by Carrasco et al. (2020). In brief, we used iCount (https://icount.readthedocs.io/en/latest/index.html) to align iCLIP reads and call significant cross-link regions. Intronic cross-link sites were called using iCount, and the exonic signal of isoform-specific exons was removed before computing the mean coverage per region across replicates. These signal tracks represent highly confident intronic cross-link sites. For the analysis of ELAV iCLIP signals in flanking introns, we first constructed an intron database to identify sets of introns flanking circRNAs and differentially expressed exons. This enabled us to compute signal enrichment in distinct groups. To create the intron database, we selected protein-coding genes, excluded first/last exons from alternative transcription start sites (TSSs) and alternative transcription end sites (TESs), and discarded nonexpressed exons. The remaining exons were then subtracted from gene coordinates, resulting in the intron database. We excluded introns <10 nt. To compute iCLIP enrichment, we selected the following genomic feature sets: (1) upstream and downstream introns immediately flanking a circRNA, (2) nonflanking introns, and (3) all introns of the gene set. To assign introns to circRNAs, we collapsed overlapping circRNAs whose respective 5′ and 3′ ends lie within 10 nt of each other. Introns were assigned to a feature if they were within a 10 nt window. For each set of introns, we assessed enrichment by comparing the normalized proportions of cross-linked sites adjusted for intron length. We constructed a contingency table using the length-normalized counts of cross-linked sites and performed a χ^2^ test to determine whether the observed proportions in flanking introns were significantly enriched compared with nonflanking introns. The iCLIP profile represents the piled-up count of cross-link sites within normalized intron sizes. The code to compute enrichments from iCLIP data is available at https://github.com/hilgers-lab/circles2024/iCLIP.intron_analysis. To identify motifs enriched in ELAV iCLIP signal, BED files were submitted to the MEME suite server using the dm6 genome. Known motif enrichment was assessed using SEA (Bailey and Grant 2021), and novel motif discovery was performed using STREME (Bailey 2021).

### Detection of differential alternative splicing events

To identify alternative splicing events, rMATS (Shen et al. 2014) was performed using the snakePipes mRNA-seq pipeline, adding the flag “—rMATS.” Splicing events were classified as neuronal if, when comparing neuronal and nonneuronal cell populations, the following applied: FDR < 0.01 and IncLevelDifference > 0.1. Parameters for the definition of ELAV-dependent splicing were FDR < 0.01 and abs(IncLevelDifference) > 0.1. To identify splicing events in the *Glut1* gene, duplicated reads were removed from the BAM file after alignment using the view function in SAMtools with the parameters “samtools view -b -F 0 × 400.” Subsequently, reads spanning different exon junctions were counted using featureCounts (Liao et al. 2014) with the parameters “featureCounts -F SAF -p ‐‐primary -B -Q 1 -s 2 -J -C –countReadPairs.” The contribution of each exon junction to *Glut1* gene expression was calculated by normalizing the junction read count against the total read count mapped to *Glut1* exons.

### Calculation of splicing efficiencies from nascent RNA-seq data

To calculate splicing efficiencies, we used previously published nascent RNA-seq data and a modified quantification method (Khodor et al. 2012). First, we generated a reference annotation by selecting the last nucleotides of each intron; i.e., the region covering the last (3′) 30% of each intron's length. For the downstream exon, the quantification used the nucleotides constituting the first (5′) 30% of the exon's length. Reads aligned to either the intron or the exon were counted using the summarizeOverlaps function from the GenomicAlignments package (Lawrence et al. 2013). These reads were then normalized using DEXseq (Anders et al. 2012). To quantify splicing efficiencies, we calculated the ratio of reads in introns to reads in exons and subtracted this value from 1. Values closer to 1 indicate a higher splicing efficiency (“faster splicing”), as they represent a lower amount of intron reads relative to exon reads. Values closer to 0 indicate lower splicing efficiencies (“slower splicing”). The methodology and code for this analysis are available at https://github.com/hilgers-lab/SpliceFlow.

### Prediction of RCMs within flanking intron sequences

To identify RCMs, we used the method described by Ivanov et al. (2015). The procedure involved a BLAST alignment for each pair of introns flanking a circRNA to identify all the potential candidates. The parameters that we used for the alignments were “-strand minus -word_size 7 -outfmt 0.” RCMs >20 nt in length were used for downstream analysis. To calculate the conservation score for RCMs, the phastCons conservation score for each genomic location was obtained using the GenomicScores package with the function getGScores(“phastCons27way.UCSC.dm6”). The conservation scores for RCMs were calculated using the gscores function with the parameter “summaryFun = mean” (Siepel et al. 2005).

### Overlap of RCMs with genomic repeats

Interspersed repeats, including transposable elements from the dm6 genome, were obtained from RepeatMasker (http://www.repeatmasker.org) and analyzed for overlap with genomic regions. Permutation tests were conducted using the permTest function from the regioneR package (Gel et al. 2016) to statistically assess overlap levels. The test parameters used were ntimes = 500, randomize.function = randomizeRegions, evaluate.function = numOverlaps, and genome = all.gene.

## Supplemental Material

Supplement 1

Supplement 2

Supplement 3

Supplement 4

Supplement 5

Supplement 6

Supplement 7
