# Supplementary material for: ELAV mediates circular RNA biogenesis in neurons
Source: Genes Dev. 2025 Sep 1;39(17-18):1064–80. doi: 10.1101/gad.352670.125 (PMC12404201; doi:10.1101/gad.352670.125)
Supplement: Supplement 1 [file Supplemental_Data.pdf]

Supplemental Tables legend

Table S1. CircRNA identification and quantification in flow-sorted cell populations from Drosophila embryos and adult heads. Related to Fig. 1 and Fig. 2. circRNAs identified as differentially expressed in each indicated dataset (neurons vs. non-neurons at 14–16h, neurons vs. non-neurons at 18–20h, *Δelav* neurons vs. wild-type neurons at 14–16h, *ΔelavΔfne* neurons vs. wild-type neurons at 18–20h, *ΔfneΔrbp9* vs. wild-type adult heads), with quantification results.

Table S2. xRIP-seq identification of transcripts directly bound by ELAV. Related to Fig. 3. Transcripts identified as enriched in each indicated dataset (anti-ELAV antibody xRIP vs. input in wild-type flies, and anti-Flag antibody xRIP vs. input in $elav^{FLAG}$ flies), with quantification results.

Table S3. Identification and quantification of alternatively spliced exons in flow-sorted cell populations from Drosophila embryos. Related to Fig. 4. Exons identified as differentially expressed in each indicated dataset (neuron-specific splicing events defined in neurons vs. non-neurons at 18–20h, alternative splicing events defined in *ΔelavΔfne* neurons vs. wild-type neurons at 18–20h), with quantification results.

Table S4. Genes that undergo ELAV-dependent AS, APA, or circRNA formation. Related to Fig. 4. Genes containing differentially expressed exons (ELAV-dependent AS) or hosting differentially expressed circRNAs (ELAV-dependent circRNA) in *ΔelavΔfne* neurons vs. wild-type neurons at 18–20h. Also listed are genes previously identified (Carrasco et al. 2022) as undergoing ELAV-dependent alternative polyadenylation (APA). Overlaps between the gene groups are indicated.

Table S5. Relative contribution of indicated splicing events in the gene *Glut1*, in control or $Glut1^{\Delta RCM}$ embryos. Related to Fig. 5. In two different $Glut1^{\Delta RCM}$ mutants (18-20h embryos), junction counts for each combination of 5' and 3' splice sites are normalized to expression of the gene.

Table S6. Recombinant DNA and RT-qPCR oligonucleotides used in this study.

Supplemental Figures and figure legends

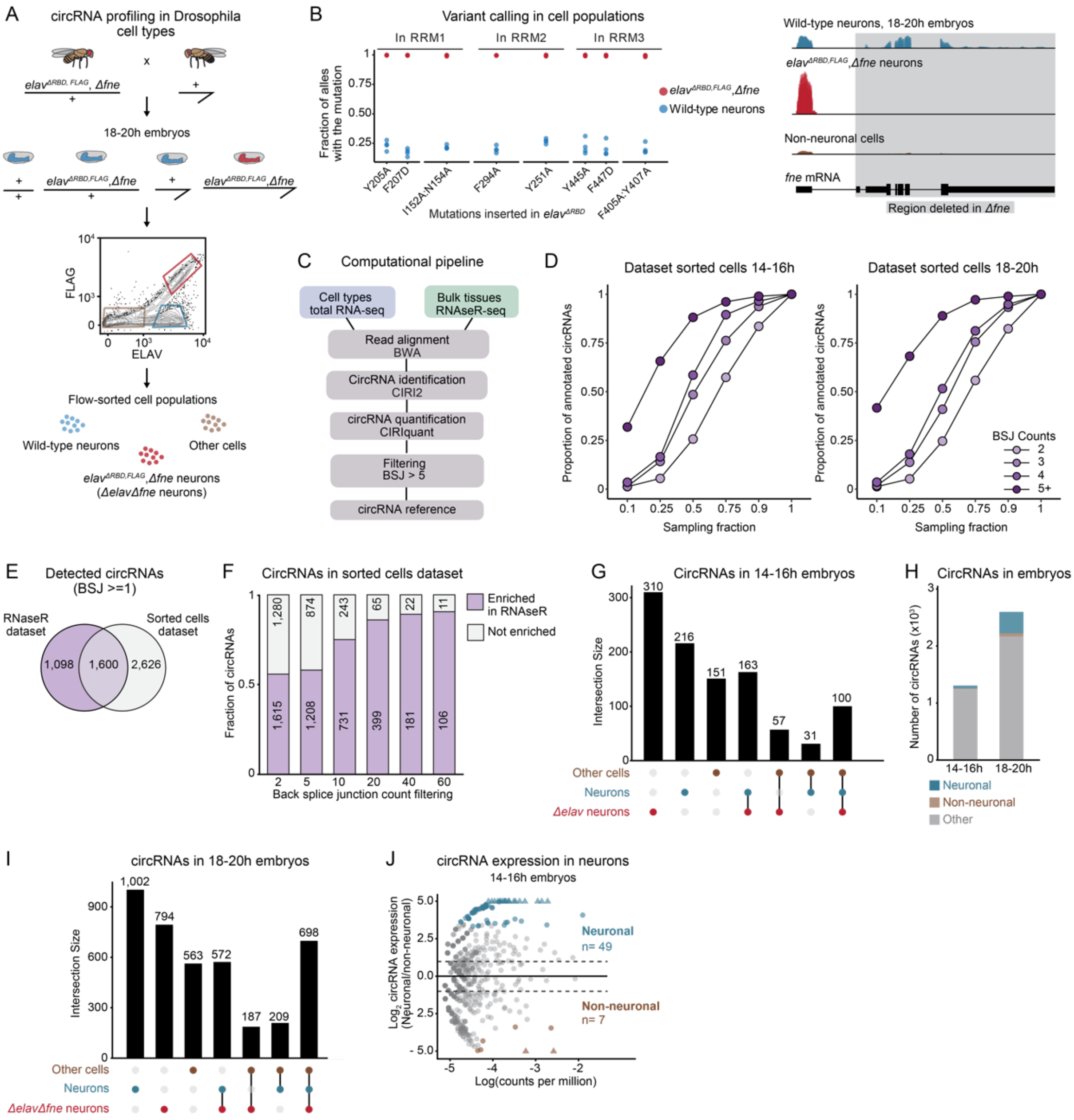

Figure S1. Landscape of neuronal circRNAs in Drosophila embryos. Related to Figure 1.

(A) Experimental overview: cells from embryonic progeny of *elav^{RBD,FLAG},Δfne* heterozygous flies (carrying a Flag-tagged *elav* RNA-binding dead allele recombined with an *fne* null mutation) were FACS-sorted into three distinct populations: wild-type neurons (ELAV+, Flag-), *ΔelavΔfne* mutant neurons (ELAV+, Flag+), and non-neuronal cells (ELAV-, Flag-). Total RNA-seq was performed on these cell populations in biological replicates. circRNA expression was quantified from total RNA-seq data, measuring reads spanning the back-splice junction unique to circRNAs.

(B) Validation of loss-of-function mutation of *elav* (left) and *fne* (right) in the *ΔelavΔfne* mutant neuron population. Left, variant calling in total RNA-seq data in the indicated cells. Shown is the fraction of reads carrying each indicated mutation, compared to the total number of reads overlapping the mutated

region. Each dot represents one sample replicate. Right, total RNA-seq tracks at the *fne* locus in the sorted cell populations. The loss of *fne* signal in *ΔelavΔfne* mutant neurons (downstream of the first exon) is highlighted.

(C) Computational pipeline to build a circRNA reference annotation. For each dataset, reads that overlap back-splice junctions (BSJs) were used to annotate circRNAs using the CIRI2 package (Gao et al., 2020) and quantified using CIRIquant (Zhang et al., 2020). Only circRNAs with ≥5 BSJ counts were considered for the reference annotation.

(D) Saturation analysis of circRNA detection in the indicated RNA-seq datasets, grouped by their expression in BSJ counts. Reads were randomly sampled in the indicated fractions and the circRNA annotation pipeline (omitting the ≥5 BSJ filtering step) was performed in each fraction.

(E) Venn diagram showing the number of circRNAs detected in the indicated RNA-seq datasets before BSJ filtering. Data from replicates and from cell populations obtained from the same embryo time point were pooled.

(F) Proportion of circRNAs enriched in the RNase R dataset, grouped by the number of BSJ counts used for filtering. BSJ≥5 was used as a cutoff for subsequent analyses.

(G) Overlap of circRNAs detected across cell populations sorted from 14–16h embryos.

(H) Number of circRNAs with neuron-specific expression (neuronal, non-neuronal, other) in cell populations sorted from 14–16h and 18–20h embryos. circRNAs were considered significantly enriched in neurons compared to non-neuronal cells (neuronal) if $p<0.05$, Log(CPM)>-5.4 and $Log_2FC \geq 1$. circRNAs were considered significantly depleted (non-neuronal) if $p<0.05$, Log(CPM)>-5.4 and $Log_2FC \leq -1$.

(I) Overlap of circRNAs detected across cell populations sorted from 18–20h embryos.

(J) Differential circRNA expression in neurons compared to non-neuronal populations represented as a function of BSJ counts per million. Significantly enriched (neuronal) circRNAs ($p<0.05$, Log(CPM)>-5.4, $Log_2FC \geq 1$, blue) and depleted (non-neuronal) circRNAs ($p<0.05$, Log(CPM)>-5.4 and $Log_2FC \leq -1$, brown) are highlighted.

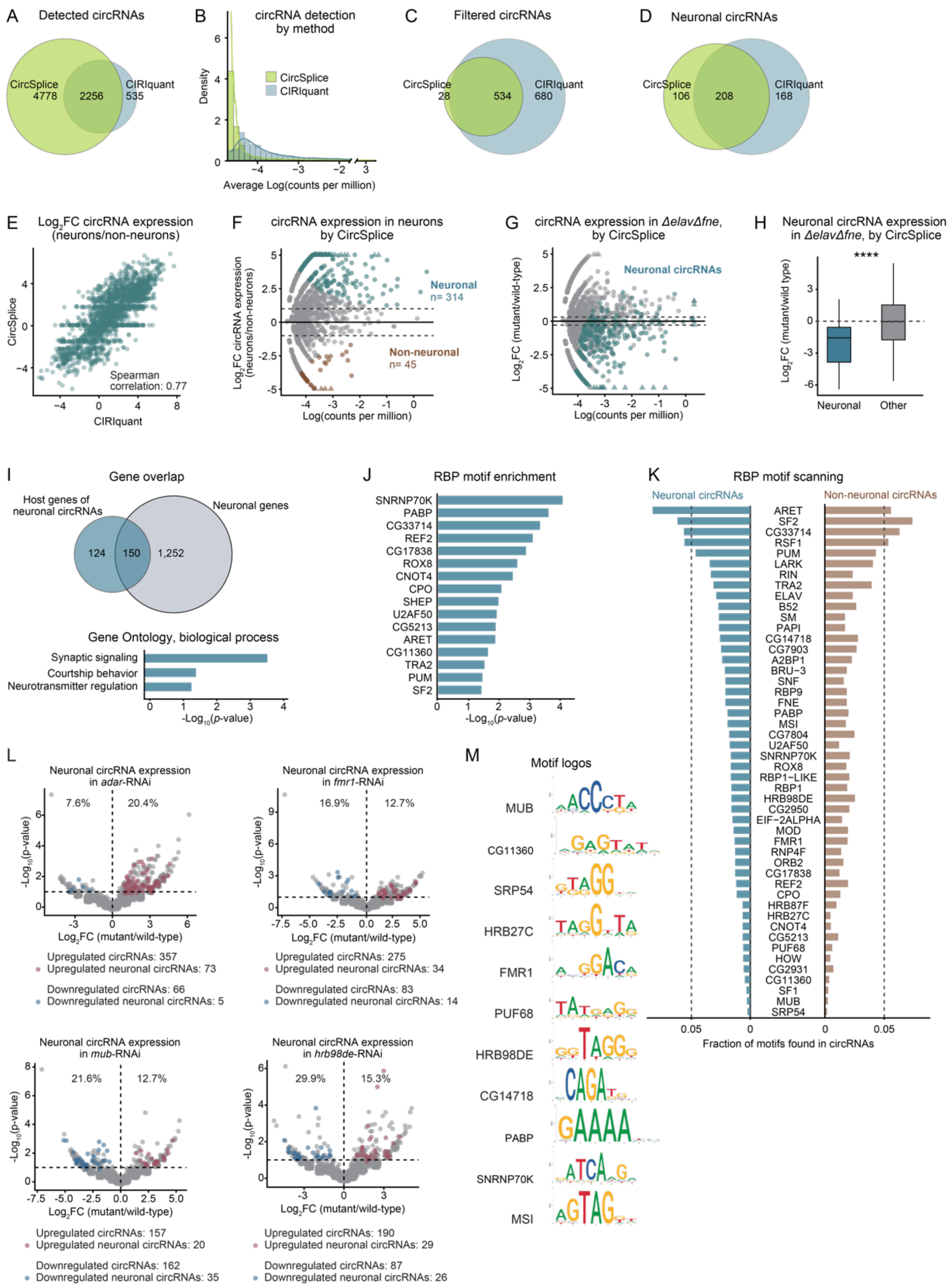

Figure S2. ELAV regulates neuronal circRNA expression. Related to Figure 1 and Figure 2.

(A) Venn diagram showing the number of circRNAs identified by circSplice and CIRIquant, respectively.

(B) Histogram showing circRNA density in function of BSJ read count (binned), quantified from total RNA-seq data using either CircSplice or CIRIquant. BSJ reads are represented as average for three populations of the 18–20h time point (neurons, non-neurons and *ΔelavΔfne* mutant neurons).
(C) Venn diagram showing the number of circRNAs filtered by BSJ read count (Log(CPM)>-4) identified by either CircSplice or CIRIquant.
(D) Venn diagram showing the number of neuronal circRNAs ($p<0.05$ and Log(CPM)>-5.4, $Log_2FC \geq 1$), identified by either CircSplice or CIRIquant after filtering.
(E) Differential circRNA expression in neurons compared to non-neuronal populations calculated by CircSplice, in function of differential expression calculated by CIRIquant. Each dot represents a circRNA identified by both methods.
(F) Differential circRNA expression in neurons compared to non-neuronal populations, calculated using CircSplice, represented as a function of BSJ counts per million. Significantly enriched (neuronal) circRNAs ($p<0.05$ and Log(CPM)>-5.4, $Log_2FC \geq 1$, blue) or depleted (non-neuronal) circRNAs ($Log_2FC \leq -1$, brown) are highlighted.
(G) Differential circRNA expression in wild-type neurons compared to *ΔelavΔfne* neurons, calculated using CircSplice, represented as a function of BSJ counts per million. Highlighted dots represent circRNAs classified as neuronal (Panel F: 314 neuronal circRNAs). The dotted line indicates the abs(Log_2FC)≥0.3 cutoff.
(H) Differential circRNA expression, calculated using CircSplice, for neuronal circRNAs and other expressed circRNAs in *ΔelavΔfne* mutant neurons compared to wild-type neurons. ****$p<0.0001$ (two-tailed Welch's t-test).
(I) Top, Venn diagram showing the number of genes that are host genes of neuronal circRNAs, and of neuronal genes. Neuronal genes are genes whose differential transcript expression was significantly higher ($p<0.01$, $Log_2FC \geq 2$) in neurons compared to non-neuronal cells in 18–20h embryonic cell populations. Bottom, Gene Ontology classification (GO terms, biological processes) of host genes of neuronal circRNAs. 203 host genes produce 1 circRNA; 71 host genes produce 2 or more circRNAs; in total, 274 host genes produce 396 circRNAs.
(J) RBP motifs significantly enriched at the BSJ (±25 nt) of neuronal circRNAs compared to other expressed circRNAs in cell populations sorted from 18–20h embryos.
(K) RBP motif scanning at the BSJ (±25 nt) of circRNAs, comparing neuronal and non-neuronal circRNAs (in cell populations sorted from 18–20h embryos).
(L) Volcano plot showing differentially expressed circRNAs in RBP-RNAi treated adult fly brains, compared to control (GFP-RNAi). Highlighted dots represent circRNAs classified as neuronal (Fig. 1D, 376 neuronal circRNAs) that were significantly upregulated (red, p-value<0.1 and $Log_2FC>1$) or downregulated (blue, p-value<0.1 and $Log_2FC<-1$). Dots are jittered to reduce overlap. To generate these plots, RNA-seq data from GSE126631 (Sapiro et al., 2020) were analyzed using CIRIquant.
(M) Sequence logos of the binding motifs for the indicated RBPs shown in Fig. 1E.

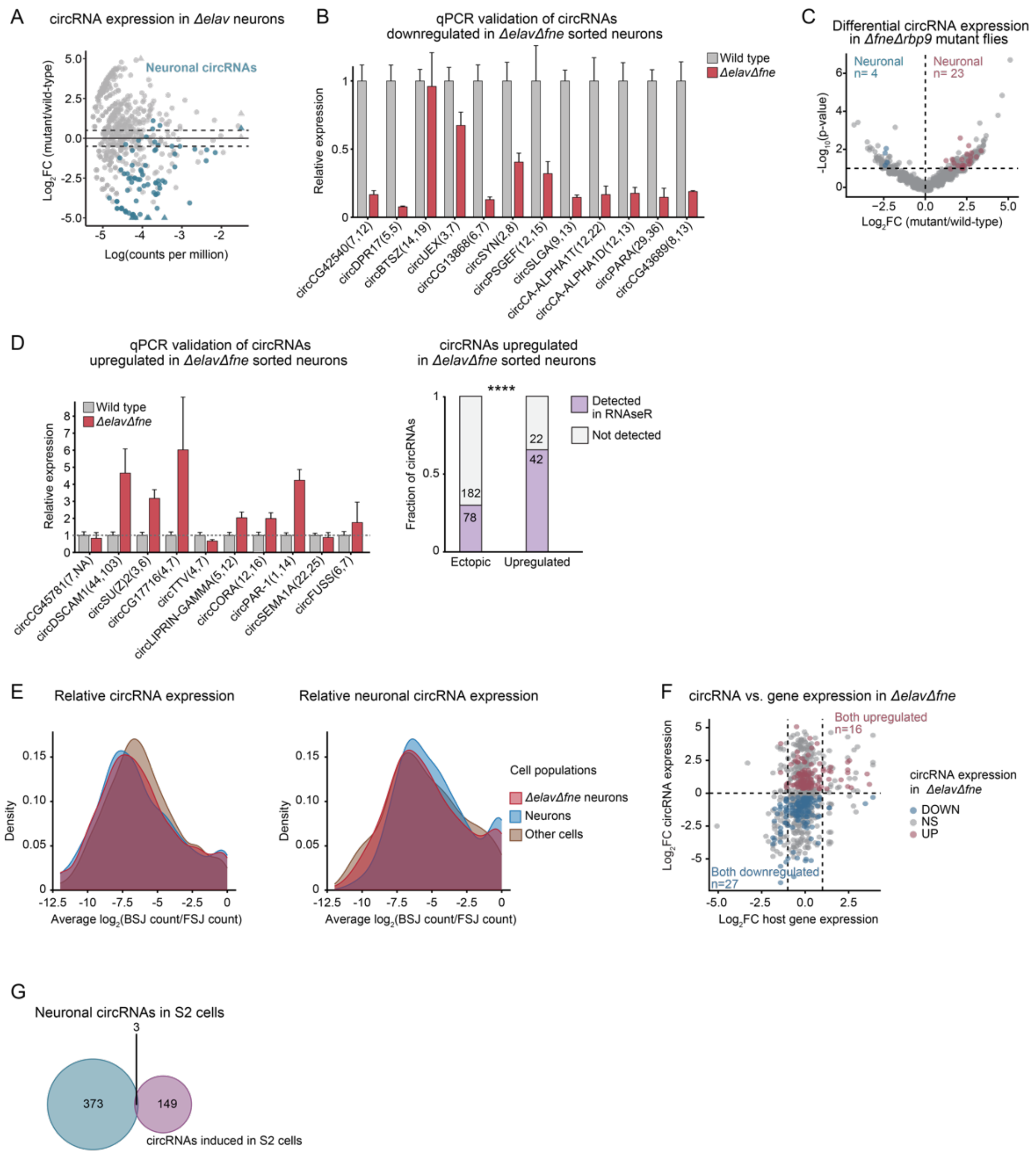

Figure S3. ELAV regulates neuronal circRNA expression. Related to Figure 1 and Figure 2.

(A) Differential circRNA expression in wild-type neurons compared to *Δelav* neurons, represented as a function of BSJ counts per million. Highlighted dots represent circRNAs classified as neuronal in 14–16h embryos (Fig. S1J, 49 neuronal circRNAs). The dotted line indicates the abs(Log_2FC)≥0.5 cutoff used to classify a circRNA as affected in *Δelav*.

(B) RT-qPCR validation and quantification of circRNAs downregulated in *ΔelavΔfne* mutant embryos. circRNA levels were normalized to *Rpl32* (*rp49*) mRNA and levels in control flies (wild type) were set to the value 1. Error bars represent mean ±SD of three biological replicates for each genotype. circRNAs were quantified using divergent, BSJ-spanning primers that only detect circular and not linear transcripts of the indicated genes.

(C) Volcano plot showing differentially expressed circRNAs in *ΔfneΔrbp9* adult fly heads compared to wild-type fly heads. Highlighted dots represent circRNAs classified as neuronal (Fig. 1D, 376 neuronal circRNAs) that were significantly upregulated (p-value<0.1 and $Log_2FC>1$) or downregulated (p-value<0.1 and $Log_2FC<-1$). Dots are jittered to reduce overlap. circRNAs significantly regulated were quantified as well as regulated neuronal circRNAs. To generate these plots, total-RNA-seq data from GSE183816 (Grzjeda et al., 2022) were analyzed using CIRIquant.

(D) Left, RT-qPCR validation and quantification of circRNAs downregulated in *ΔelavΔfne* mutant embryos. circRNA levels were normalized to *Rpl32* (*rp49*) mRNA and levels in control flies (wild type) were set to the value 1. Error bars represent mean ±SD of three biological replicates for each genotype. circRNAs were quantified using divergent, BSJ-spanning primers that only detect circular and not linear transcripts of the indicated genes. Right, Proportion of circRNAs that were upregulated in *ΔelavΔfne* neurons compared to wild-type neurons (ectopic or upregulated) that were detected (≥1 BSJ) in the RNaseR dataset. "Ectopic" denotes circRNAs detected exclusively in the *Δelav* or *ΔelavΔfne* cell population, and not detected in other cell types. "Upregulated" denotes circRNAs significantly upregulated in *ΔelavΔfne* neurons compared to wild-type neurons in cell populations sorted from 18–20h embryos. ****p<0.0001 (Pearson's Chi-squared test).

(E) Density plot showing distribution of all (left) and neuronal (right) circRNAs in function of Log_2(junction ratio). Junction ratio represents the relative circRNA expression compared to its cognate linear RNAs. Represented are circRNAs with at least 5 BSJs counted in combined 18–20h embryonic cell populations.

(F) Differential circRNA expression in *ΔelavΔfne* neurons compared to wild-type neurons as a function of differential host gene expression in the same data set. Highlighted dots represent circRNAs significantly regulated in *ΔelavΔfne* neurons. The dotted line indicates $Log_2FC=0$ at circRNA level and $|Log_2FC|=1$ at gene level. The number of circRNAs significantly upregulated (shown in Supplemental Table S1) with their host genes also upregulated ($Log_2FC>1$), are shown as well as circRNAs significantly downregulated (shown in Supplemental Table S1) with their host genes also downregulated ($Log_2FC<-1$).

(G) Venn diagram showing neuronal circRNAs (Fig. 1D, 376 neuronal circRNAs) and circRNAs induced in ELAV expressed S2 cells expressing ELAV (Fig. 2G, 152 induced circRNAs).

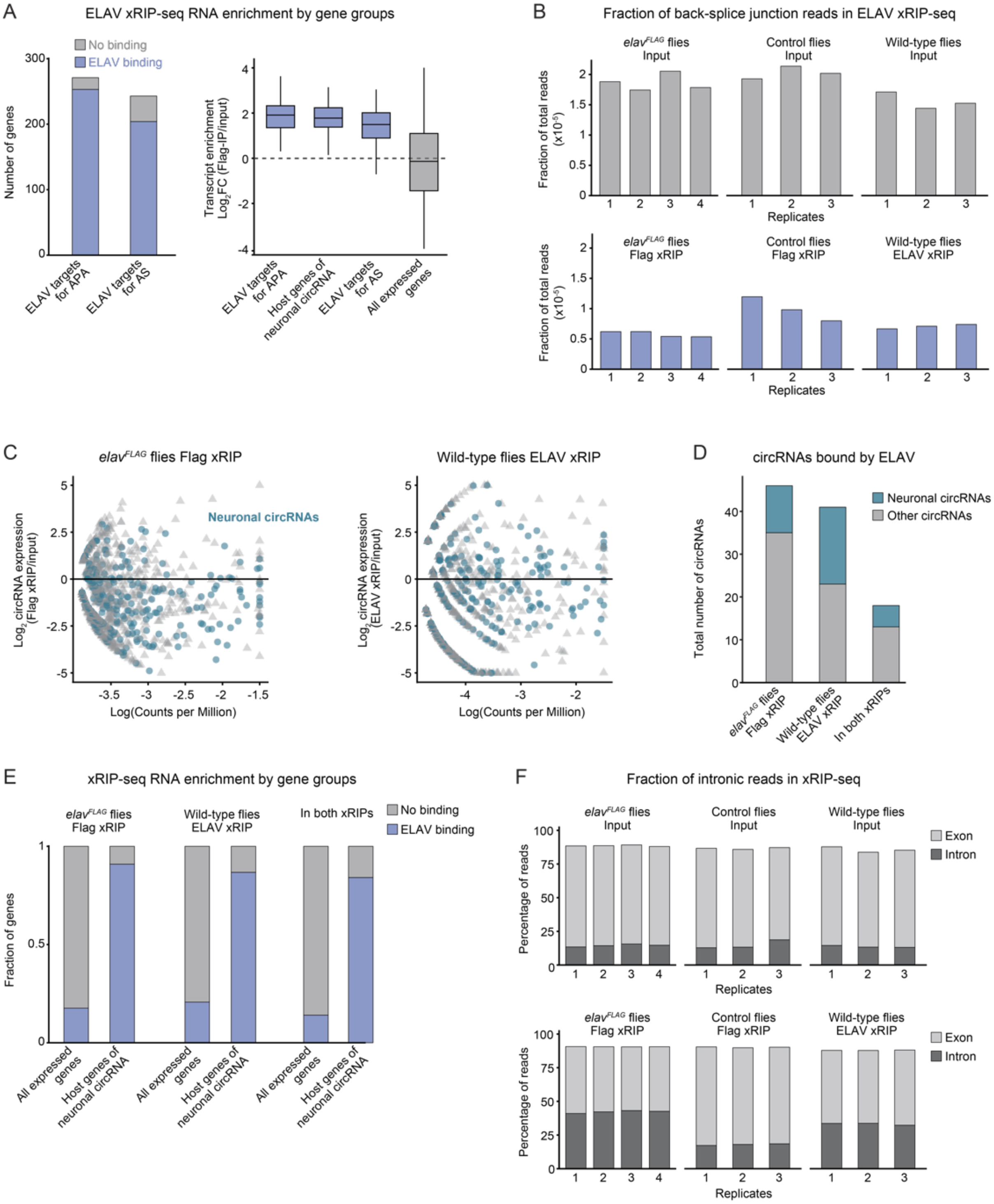

Figure S4. ELAV binds pre-mRNA of circRNA host genes. Related to Figure 3.

(A) Left, proportion of genes in each group that displayed significant transcript enrichment in Flag-ELAV xRIP-seq compared to input. “ELAV targets for APA" denotes genes identified in (Carrasco et al., 2020) as genes that undergo ELAV-dependent neuronal alternative polyadenylation. “ELAV targets for AS” denotes genes identified as genes that undergo ELAV-dependent neuronal alternative splicing. Right, ELAV binding to transcripts of the indicated gene groups, calculated as the differential transcript expression in Flag-ELAV xRIP compared to input in *elav*^{FLAG} flies. Genes were considered significantly

bound if $Log_2FC>1$ and $p<0.01$. Genes were considered expressed if they were detected in xRIP-seq and input samples at baseMean>4.

(B) ELAV binding to circRNAs, represented as fraction of total reads that overlap BSJs across replicates, in input (top) and xRIP-seq (bottom) samples in the indicated ELAV pull-down experiments (Flag IP in *elav*^{FLAG} flies, ELAV IP in wild-type flies) and the control experiment (Flag IP in wild-type flies).

(C) Left, differential circRNA expression in Flag-ELAV xRIP-seq (left) and ELAV xRIP-seq (right) compared to respective inputs, represented as a function of BSJ counts per million. Highlighted dots represent circRNAs classified as neuronal in 18–20h embryonic cell populations. Right, proportion and number of neuronal circRNAs among circRNAs that displayed significant enrichment in Flag-ELAV xRIP, ELAV xRIP, and both xRIPs. circRNAs were considered significantly bound by ELAV if BSJ expression was significantly enriched in xRIP compared to input ($Log_2FC>0.5$ and $p<0.1$).

(D) Total number of detected circRNAs across samples of xRIP-seq experiment.

(E) Proportion of genes in each gene group that displayed significant transcript enrichment in Flag-ELAV xRIP-seq compared to input in *elav*^{FLAG} flies ($Log_2FC>1$ and $p<0.01$).

(F) ELAV binding to transcript regions, represented as fraction of total reads that overlap intronic and exonic regions, respectively, across replicates, in input (top) and xRIP-seq (bottom) samples in the indicated ELAV pull-down experiments (Flag IP in *elav*^{FLAG} flies, ELAV IP in wild-type flies) and the control experiment (Flag IP in wild-type flies).

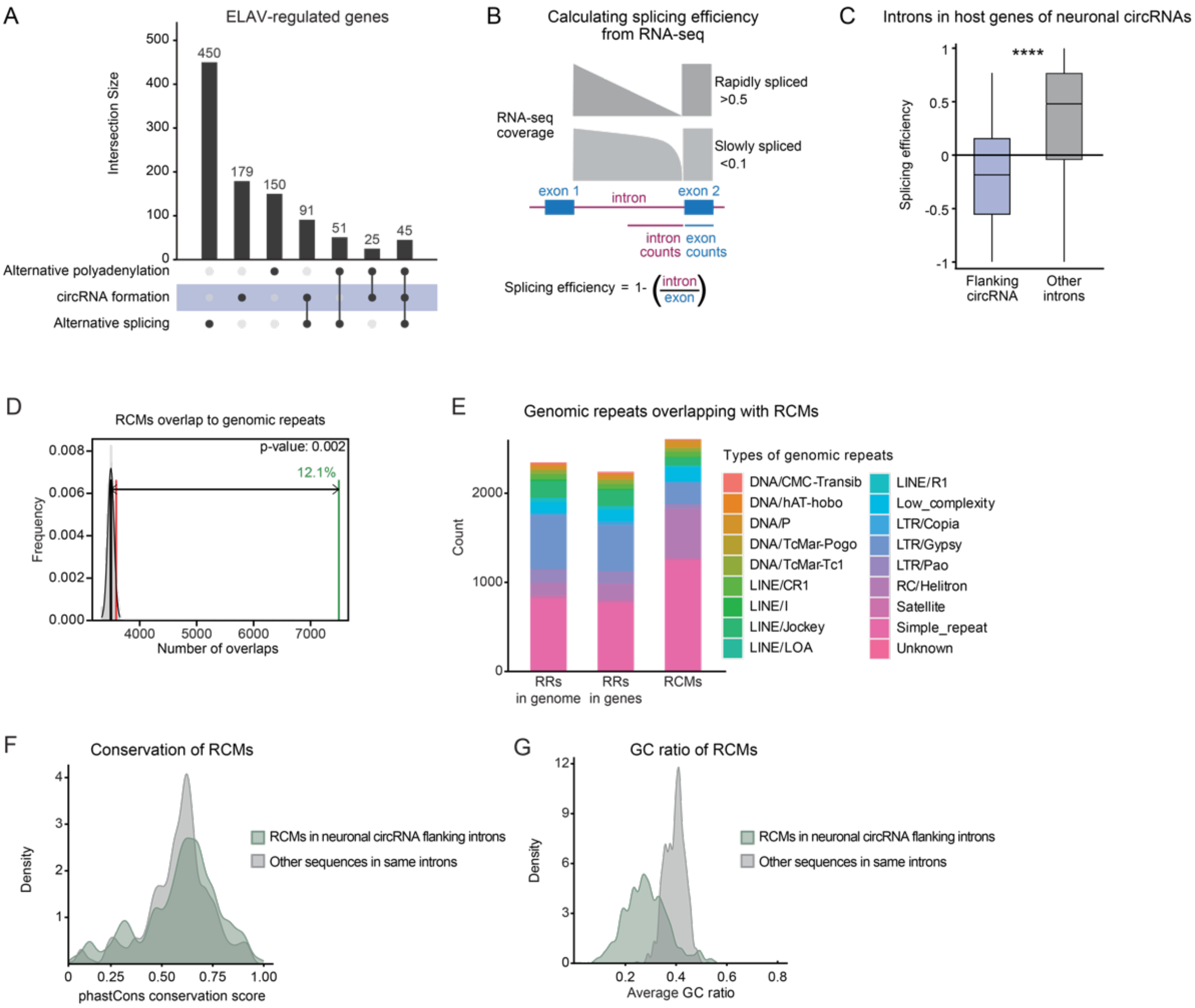

H

Top motifs in ELAV iCLIP

Motif	RBP	P-value	E-value	Q-value	Enrichment ratio
	ELAV	5.70e-41	3.82e-39	3.82e-39	1.24
	RBP9	2.04e-36	1.37e-34	6.84e-35	1.23

I

Top motifs in RCMs flanking neuronal circRNAs

Motif	RBP	P-value	E-value	Q-value	Enrichment ratio
	ROX8	2.03e-314	1.36e-312	3.41e-313	2.13
	PUM	7.24e-271	4.85e-269	8.09e-270	2.11
	CG11360	4.06e-249	2.72e-247	3.40e-248	1.91
	ELAV	6.33e-247	4.24e-245	4.71e-246	2.27

Figure S5. ELAV binds to RCMs to inhibit forward-splicing and promote back-splicing. Related to Figure 4.

(A) Upset plot showing the number and overlap of genes that undergo ELAV-dependent alternative polyadenylation, alternative splicing, and circRNA formation.

(B) Schematic representation of how splicing efficiencies were calculated. Nascent-seq data from (Khodor et al., 2011) were mapped. Reads that aligned to the last 15 nucleotides of the intron (15 nt of the intron 3' end), and reads that aligned to the first 15 nucleotides of the downstream exon (15 nt of the exon 5' end) were quantified. The differential read count in the intron region compared to the exon region was calculated. This value was subtracted from 1 to obtain the splicing efficiency. Values close to 1 represent a high splicing efficiency ("fast splicing").

(C) Splicing efficiency in genes that produce neuronal circRNAs, comparing introns that flank the neuronal circRNA's BSJ to all other introns of the same gene. ****p<0.0001 (one-tailed Welch's t-test).

(D) Plot showing result of the permutation test comparing the overlap between RCM regions with genomic repeat regions located within genes. 500 random regions located within genes and with the same size as RCM regions were tested in the permutation test. (Permutation test, times =500). The number of random regions overlapping with repeat regions is fitted with a normal distribution (grey), with p=0.05 indicated by a red line. The green line represents the number of RCMs overlapping with repeat regions, the proportion of overlaps is also shown.

(E) Number of genomic repeats within genes that overlap with either RCM regions or randomized genomic regions. RRs: Random regions. Only types with counts greater than 5 in either region set are shown.

(F) Histogram showing region density in function of phastCons conservation scores for RCMs flanking neuronal circRNAs, compared to other sequences within the same introns. For this comparison, two base pairs of the splice sites were removed from the start and end of the introns.

(G) Histogram showing region density in function of GC ratio for RCMs flanking neuronal circRNAs, compared to other sequences within the same introns. For this comparison, 2 base pairs of the splice sites were removed from the start and end of the introns. In the t-test, p<0.0001.

(H) RBP binding motifs enriched at the top 2 levels of ELAV iCLIP signal peaks compared to shuffled sequences.

(I) RBP binding motifs enriched in RCMs surrounding neuronal circRNAs, compared to other sequences within the same introns. Motifs with only As or/and Ts are not shown.
